# An integrative mechanistic model of thymocyte dynamics

**DOI:** 10.3389/fimmu.2024.1321309

**Published:** 2024-02-26

**Authors:** Victoria Kulesh, Kirill Peskov, Gabriel Helmlinger, Gennady Bocharov

**Affiliations:** ^1^ Research Center of Model-Informed Drug Development, I.M. Sechenov First Moscow State Medical University, Moscow, Russia; ^2^ Marchuk Institute of Numerical Mathematics of the Russian Academy of Sciences (RAS), Moscow, Russia; ^3^ Modeling & Simulation Decisions FZ - LLC, Dubai, United Arab Emirates; ^4^ Sirius University of Science and Technology, Sirius, Russia; ^5^ Biorchestra US Inc., Cambridge, MA, United States; ^6^ Institute for Computer Science and Mathematical Modelling, I.M. Sechenov First Moscow State Medical University, Moscow, Russia; ^7^ Moscow Center of Fundamental and Applied Mathematics at INM Russian Academy of Sciences (RAS), Moscow, Russia

**Keywords:** immune system, thymus, thymopoiesis, thymic involution, mechanistic model

## Abstract

**Background:**

The thymus plays a central role in shaping human immune function. A mechanistic, quantitative description of immune cell dynamics and thymic output under homeostatic conditions and various patho-physiological scenarios are of particular interest in drug development applications, e.g., in the identification of potential therapeutic targets and selection of lead drug candidates against infectious diseases.

**Methods:**

We here developed an integrative mathematical model of thymocyte dynamics in human. It incorporates mechanistic features of thymocyte homeostasis as well as spatial constraints of the thymus and considerations of age-dependent involution. All model parameter estimates were obtained based on published physiological data of thymocyte dynamics and thymus properties in mouse and human. We performed model sensitivity analyses to reveal potential therapeutic targets through an identification of processes critically affecting thymic function; we further explored differences in thymic function across healthy subjects, multiple sclerosis patients, and patients on fingolimod treatment.

**Results:**

We found thymic function to be most impacted by the egress, proliferation, differentiation and death rates of those thymocytes which are most differentiated. Model predictions also showed that the clinically observed decrease in relapse risk with age, in multiple sclerosis patients who would have discontinued fingolimod therapy, can be explained mechanistically by decreased thymic output with age. Moreover, we quantified the effects of fingolimod treatment duration on thymic output.

**Conclusions:**

In summary, the proposed model accurately describes, in mechanistic terms, thymic output as a function of age. It may be further used to perform predictive simulations of clinically relevant scenarios which combine specific patho-physiological conditions and pharmacological interventions of interest.

## 1 Introduction

The thymus is a primary lymphoid organ in the chest cavity, where immature T cells, known as thymocytes, differentiate into functional T cells. Structurally, the thymus is surrounded by a fibrous capsule and consists of multiple lobes which are separated into two main regions, the outer cortex and the inner medulla (1). According to their developmental origin, thymic cells are subdivided into hematopoietic cells (CD45-positive) and stromal cells (CD45-negative); the non-hematopoietic components are further divided into cortical and medullary thymic epithelial cells (cTEC and mTEC, respectively) and various mesenchymal cells (fibroblasts, capsule- and septae-forming connective tissue cells, as well as endothelial cells) (2). Functionally, the thymus comprises the true thymic epithelial space (TES), where thymopoiesis occurs, and the non-epithelial, non-thymopoietic perivascular space (3).

The T-cell developmental process starts when T-cell precursors from the bone marrow transfer into the thymus via blood vessels at the corticomedullary junction. T-cell precursors are also known as bone marrow-derived thymus-seeding progenitors (TSPs) and most likely comprise multiple cell types: IL-7R+ common lymphoid progenitors (CLPs), Flt3+ lymphoid primed multipotent progenitors (LMPPs) and other T-cell precursors (4). Once in the thymus, the T-cell developmental process proceeds with two phases: 1. Initiation of antigen receptor gene rearrangement and β-selection; 2. Positive and negative selection and further differentiation to specific effector cell lineages, *i.e.*, CD4+ helper T cells, CD8+ cytotoxic T cells, or CD4+ regulatory T cells (1).

TSPs proliferate in the subcapsular cortex and differentiate into double negative (DN) thymocytes. Further DN differentiation includes several sequential cellular sub-types: DN1, DN2, DN3 and DN4, differing by various cell surface markers and related functions, *i.e.*, DN1 thymocytes (CD177+ CD44+ CD25- CD4- CD8-) migrate to the cortex and actively proliferate; DN2 thymocytes (CD177+ CD44+ CD25+ CD4- CD8-) rearrange their γ-, δ-, and β-chains of T-cell receptor (TCR); DN3 thymocytes (CD177+ CD44- CD25- CD4- CD8-) perform β-selection, whereby cells failing to produce a functional TCR β-chain undergo apoptosis (5). Those cells that successfully complete β-selection initiate rearrangement of the TCR α-chain, yielding DN4 thymocytes (CD177- CD44- CD25- CD4- CD8-), which may further differentiate into a double-positive CD4+ CD8+ (DP) phenotype. DP thymocytes undergo a two-stage selection process in the thymic cortex. Positive selection is responsible for survival of cells whose T-cell receptor can bind major histocompatibility complexes (MHC) I or II, with at least a weak affinity. During negative selection, thymocytes that bind self-peptides or MHC with high affinity undergo apoptosis. Successfully selected DP cells further differentiate into single-positive (SP) cells, which express either CD4 (CD4+ CD8-) or CD8 (CD4- CD8+) markers. SP cells reside in the thymic medulla and, upon a final maturation stage, leave the thymus and migrate as recent thymic emigrant (RTE) cells to peripheral lymphoid tissues such as the spleen, gut, and lymph nodes. In secondary lymphoid organs, RTE cells differentiate into naïve cells, with a full potential to initiate an immune response.

It is well-known that the efficiency of the T-cell developmental process in the thymus is crucial for the ability of the immune system to perform its function in controlling antigenic homeostasis, e.g., to prevent the host from severe forms of infections or cancers. This function gradually decreases with age, starting during the first year of life (3, 6). Unfortunately, the exact mechanisms underlying this process are still poorly understood (7). Several hypotheses of thymus involution have been proposed, including the aging of hematopoietic progenitors, dysfunctioning in the thymic microenvironment, or elevation in the levels of sex hormones (8, 9). It is also known that the expansion of the perivascular space - mainly due to adipogenesis and shrinkage of the thymic epithelial space - results in a decreased thymic output (3, 6). Moreover, the cortex involutes more rapidly than the medulla, according to experimental findings (10).

An integrative mathematical modeling of T-cell development may allow for a better quantitative understanding of mechanisms affecting thymocytes dynamics and the ability of the immune system in fighting infections. Several models of thymopoiesis, using various modeling techniques, have been developed in the literature (4). These models may be categorized into three types:

- Models of thymocyte population dynamics formulated with linear ordinary differential equations (ODE) (11–14). These models describe, mathematically, basic cellular turnover processes, such as proliferation, differentiation, and death.
- Models based on the logistic growth equation, to account for a maximal carrying capacity of certain structural niches in thymus, for specific cell populations (13) and/or for the total number of thymocytes (13, 15). Such models typically consider the descriptor of growth control as an important physiological component in mathematical modeling, as it limits excessive, non-physiological cell proliferation.
- ODE-based generation-structured models, which account for cell number dynamics at each stage of cell division (16).

However, there is no general agreement on an optimal model structure, to adequately describe thymus dynamics (4). Moreover, the majority of existing models feature definite limitations associated with physiological background and constraints underlying the described processes, such as cell proliferation dynamics, realistic anatomical and physiological descriptors of a niche, etc.

Similarly to thymopoiesis, various modeling approaches have been published to quantitatively describe thymus involution. In one approach, thymus function decline was modeled phenomenologically by decreasing the overall thymic output proportionally to the TES volume (17). In another approach, TES involution with age was modeled using a modified exponential decay function (6). While it is not possible to directly measure thymic function, various surrogate measures have been used, such as signal joint TCR excision circles (signal joint TRECs), to assess thymic output, or the level of expression of the proliferation marker Ki67, to assess the T-cell proliferation rate (18). Thymus involution has also been described using an age-dependent function of TES as a carrying capacity of the thymus (15). In yet another approach, exponentially decreasing proliferation rates for each modeled cell population were utilized (19). Although these modeling approaches may allow one to predict thymic output as a function of age, the respective models do not consider the effects of age on thymocyte populations (6, 18) and/or lack a solid biological basis for mechanistically describing the thymus function, since there are no experimental data on the age-related decrease in proliferative activity for each thymocyte population (19).

Historically, research on thymopoiesis dynamics has been hampered by a lack of longitudinal data on specific thymocyte types in humans; a majority of physiological studies have indeed been conducted in mouse. Challenges relating to an adequate translation or scaling of data from mouse to human are still unresolved. In general, conventional empirical models for cross-species translation are oversimplified and not suitable to address practical questions related to, for example, preclinical-to-clinical drug development. In the modern paradigm of model-informed drug discovery and development (MID3), the value of integrative, mechanistic, quantitative systems pharmacology (QSP) models has been well-recognized (20). QSP models are based on a mechanistic and quantitative description of operating biological and patho-physiological processes, with an opportunity to integrate experimental data over multiple scales of biological organization (20). Importantly, mechanistic QSP models of the immune system have been used to support decisions not only in basic research, but also in regulatory assessments. For instance, regulatory approval of the first CAR-T cell product, tisagenlecleucel, was supported by a mechanistic model describing murine immune responses to a lymphocytic choriomeningitis virus and characterizing the expansion and persistence of tisagenlecleucel, as a substitute for traditional, semi-empirical compartmental pharmacokinetic modeling (21–23).

The primary objective of the present study was to develop a mechanistic mathematical model of thymopoiesis which integrates a substantial amount of realistic, physiologically-relevant, biological details which are most crucial to thymopoiesis, such as thymus spatial structure, specific physiological cell niches, physiological homeostasis, and age-related thymus involution. An integrative model of this nature is expected to describe thymocyte numbers at quasi steady-state and to address issues relevant to the development and efficacy evaluation of novel medicines – for example, the observed changes in immune homeostasis under pathological conditions and specific effects of therapeutic interventions that modulate thymic function.

The paper is organized as follows: Section 2 describes the model development workflow, with an overview of relevant experimental data and details of the model equations used. Section 3 presents model calibration and validation results, a sensitivity analysis, and novel predictions on thymic function. Finally, Sections 4 to 5 provide, respectively, a discussion and conclusions.

## 2 Materials and methods

### 2.1 Data

#### 2.1.1 Experimental and clinical data

A systematic review of the literature has been performed, to identify all relevant sources with experimental and clinical data. The PubMed database and Google Scholar were searched using the keywords “thymocytes”, “thymus”, “human” and “flow cytometry” to identify pertinent data sources. Quantitative information on dynamic and kinetic characteristics of various thymocyte sub-populations, as well as thymic weight and cellularity during age-dependent involution was assessed from peer-reviewed articles presenting clear tables, figures, and experiments of adequate quality, ensuring the validity of the published cell phenotypes. Additionally, references from listed review papers were examined. Multiple sets of mouse and human data spanning diverse age groups were thus curated and used for model parameter estimation and model validation (**Table 1**).

**Table 1:** Experimental and clinical data used for model development.

Data description	Data assignment	Ref.
Human thymus wet weight	Parameter calibration	(6, 24)
	Model validation	(6)
Relative proportions of human double-negative, double-positive and single-positive thymocytes	Parameter calibration	(25–31)
	Model validation	(25–33)
Volumes of lymphatic tissue and of lymphocytic perivascular space in human thymus, relative proportions of thymic epithelial space	Parameter calibration	(3, 6, 34, 35)
Human thymic cortico-medullary ratio	Parameter calibration	(10)
Cell count and residence time of different thymocyte sub-types in mouse and human	Parameter calibration	(36–39)
Cell count ratio, mouse-to-human	Parameter calibration	(40)
Turnover rate of different thymocyte sub-types in mouse	Parameter calibration	(41–43)
Percentage of DN3 thymocytes that undergo apoptosis at the β-selection checkpoint	Parameter calibration	(5)
SP thymocyte emigration time in mouse	Parameter calibration	(44)
Thymocyte outflux from thymus in human	Parameter calibration	(45)
Thymic cellular density in human (cells/g)	Model validation	(25)
Human thymus volume	Model validation	(6)
Relative volume of thymic cortex in human	Model validation	(6)

#### 2.1.2 Physiological parameter ranges

Physiological ranges of DN, DP and SP thymocytes were derived based on the curated data, for model calibration and validation purposes. The total thymocyte count was calculated based on thymus wet weight and thymic cell density per gram of thymus tissue, among individuals and across an age range (6, 25). As data for each thymocyte population were limited, model calibration was performed on the 0-to-1 year old group data. Thymocyte counts for each cell population (DN, DP, SP4, and SP8 cells) in the 0-to-1 year old group were derived by multiplying the total thymocyte count range by the median relative proportion of each thymocyte population for the respective age group (25–31). Details on data transformation and related calculations are summarized in the **Supplementary Materials** (**Supplementary Tables 1**, **2**).

### 2.2 Model formulation

#### 2.2.1 Model structure

**Figure 1:**
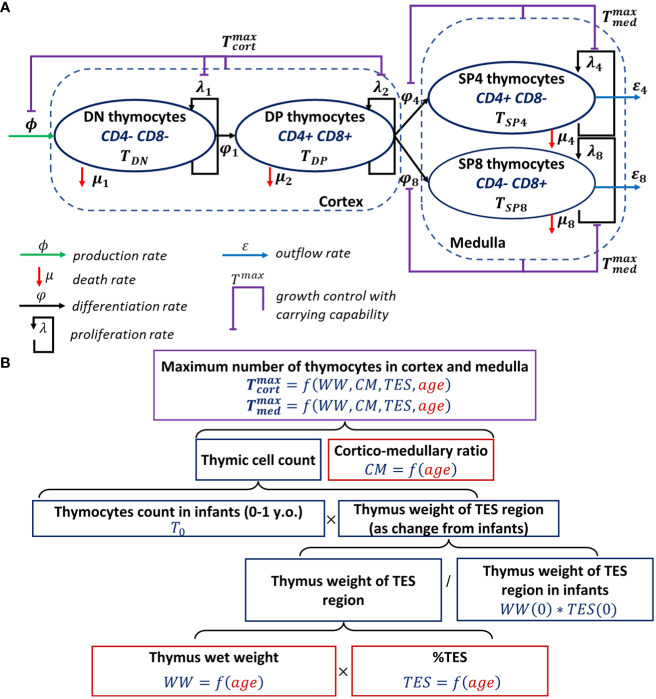
Scheme of the mechanistic model describing human thymocyte dynamics. **(A)** Model scheme, thymocyte homeostasis; **(B)** Model development steps describing thymus involution.

The schematic structure of the human thymocyte dynamic model that characterizes DN (CD4- CD8-), DP (CD4+ CD8+), SP4 (CD4+ CD8-) and SP8 (CD4- CD8+) thymocyte homeostasis is shown in **Figure 1A**. The respective time-dependent cell populations which are considered in the model are denoted as $T_{DN}\left(t\right)$ , $T_{DP}\left(t\right)$ , $T_{SP4}\left(t\right)$ and $T_{SP8}\left(t\right)$ .

The model was initially built based on a model proposed by Ye et al. (15). The inflow to the DN compartment represents thymic-seeding progenitors, which enter the thymus from the blood. The death rates of cells represent net apoptosis due to T-cell commitment and β-selection for the DN population, as well as positive and negative selections for the DP population. A death rate for SP cells was also included in the model, due to the negative selection that occurs at the SP stage, yet at a slower rate as compared to the overall death rate due to positive and negative selection in the cortex at the DP stage (36). Cell proliferation was implemented for each cell population (DN, DP, SP4 and SP8) (1, 46). Distinct maximal allowable numbers of thymocytes in the cortex and medulla were introduced, to prevent excessive proliferation of certain thymocytes in these areas, given that DN and DP cells primarily reside in the cortex and SP cells in the medulla. The maximal allowable number of thymocytes in the medulla was also set to reflect the differentiation process of DP into SP cells and the subsequent migration of cells into the medulla. In contrast to the Ye et al. structural model, we: (i) included only one compartment for DN cells; and (ii) implemented growth controls separately in the thymic cortex and medulla *vs*. the entire thymus (15, 36).

Thymus involution was modeled with an age-dependent function describing the maximal allowable number of cells in the cortex and medulla, in agreement with existing experimental data (6). To quantify such an age-dependent function, the respective data were processed as shown in the flow diagram (**Figure 1B**).

#### 2.2.2 Model equations

The mathematical model describing thymocyte dynamics consisted of a system of ordinary differential equations (ODEs), with parametrization of the growth control and age-dependent functions describing decreases in cortex and medulla cell counts.

The dynamics of DN thymocytes ( $T_{DN}$ ) was described using the following equation:


(1) $$\frac{dT_{DN}}{dt}=\phi \left(1-\frac{T_{DN}+T_{DP}}{T_{cort}^{max}\left(age\right)}\right)-\varphi_{1}T_{DN}+\lambda_{1}\left(1-\frac{T_{DN}+T_{DP}}{T_{cort}^{max}\left(age\right)}\right)T_{DN}-\mu_{1}T_{DN},$$

where $T_{cort}^{max}\left(age\right)$ represents an age-dependent function for the maximal thymocyte numbers (i.e., carrying capacity) in the thymic cortex; $\phi \left(1-\;\frac{T_{DN}+T_{DP}}{T_{cort}^{max}\left(age\right)}\right)$ represents the inflow of thymocyte precursors; $\varphi_{1}T_{DN}$ is the differentiation rate of DN cells to DP cells; $\lambda_{1}\left(1-\;\frac{T_{DN}+T_{DP}}{T_{cort}^{max}\left(age\right)}\right)T_{DN}$ represents a logistic growth function for the DN cell proliferation in the thymic cortex; $\mu_{1}T_{DN}$ represents the DN cell death; ϕ is the thymocyte precursor inflow rate (cells*d^-1^); $\varphi_{1}$ is the rate constant of DN-to-DP differentiation (d^-1^); $\lambda_{1}$ is the DN cell proliferation rate constant (d^-1^); and $\mu_{1}$ is the DN cell death rate constant (d^-1^).

The dynamics of DP thymocytes ( $T_{DP}$ ) was described by the following equation:


(2) $$\frac{dT_{DP}}{dt}=\;\varphi_{1}T_{DN}+\lambda_{2}\left(1-\;\frac{T_{DN}+T_{DP}}{T_{cort}^{max}\left(age\right)}\right)T_{DP}-\left(\varphi_{4}+\varphi_{8}\right)\left(1-\frac{T_{SP4}+T_{SP8}}{T_{med}^{max}\left(age\right)}\right)T_{DP}-\mu_{2}T_{DP},$$

where $\lambda_{2}\left(1-\;\frac{T_{DN}+T_{DP}}{T_{cort}^{max}\left(age\right)}\right)T_{DP}$ represents a logistic growth function for the DP cell proliferation in the thymic cortex; $T_{med}^{max}\left(age\right)$ represents an age-dependent function for the maximal thymocyte number (i.e., carrying capacity) in the thymic medulla; $\left(1-\;\frac{T_{SP4}+T_{SP8}}{T_{med}^{max}\left(age\right)}\right)$ is a logistic growth function in the thymic medulla; $\varphi_{1}T_{DN}$ is the DN-to-DP differentiation; $\left(\varphi_{4}+\;\varphi_{8}\right)\left(1-\;\frac{T_{SP4}+T_{SP8}}{T_{med}^{max}\left(age\right)}\right)T_{DP}$ represents the DP-to-SP4/SP8 differentiation; $\mu_{2}T_{DP}$ is the DP cell death rate; $\varphi_{1}$ is the DN-to-DP differentiation rate constant (d^-1^); $\lambda_{2}$ is the per capita DP cell proliferation rate constant (d^-1^); $\varphi_{4}$ and $\varphi_{8}$ are rate constants of DP-to-SP4 and DP-to-SP8 differentiations, respectively (d^-1^); and $\mu_{2}$ is the DP cell death rate constant (d^-1^).

The dynamics of SP4 ( $T_{SP4}$ ) and SP8 ( $T_{SP8}$ ) thymocytes were described by the following equations:


(3) $$\frac{dT_{SP4}}{dt}=\varphi_{4}\left(1-\frac{T_{SP4}+T_{SP8}}{T_{med}^{max}\left(age\right)}\right)T_{DP}-\varepsilon_{4}T_{SP4}+\lambda_{4}\left(1-\frac{T_{SP4}+T_{SP8}}{T_{med}^{max}\left(age\right)}\right)T_{SP4}-\mu_{4}T_{SP4},$$

(4) $$\frac{dT_{SP8}}{dt}=\varphi_{8}\left(1-\frac{T_{SP4}+T_{SP8}}{T_{med}^{max}\left(age\right)}\right)T_{DP}-\varepsilon_{8}T_{SP8}+\lambda_{8}\left(1-\frac{T_{SP4}+T_{SP8}}{T_{med}^{max}\left(age\right)}\right)T_{SP8}-\mu_{8}T_{SP8},$$

where $\varphi_{4}\left(1-\;\frac{T_{SP4}+T_{SP8}}{T_{med}^{max}\left(age\right)}\right)T_{DP}$ and $\varphi_{8}\left(1-\;\frac{T_{SP4}+T_{SP8}}{T_{med}^{max}\left(age\right)}\right)T_{DP}$ represent DP-to-SP4 and DP-to-SP8 differentiation rate constants, respectively; $\varepsilon_{4}T_{SP4}$ and $\varepsilon_{8}T_{SP8}$ represent SP4 and SP8 outflux rates, respectively; $\lambda_{4}\left(1-\frac{T_{SP4}+\;T_{SP8}}{T_{med}^{max}\left(age\right)}\right)T_{SP4}$ and $\lambda_{8}\left(1-\frac{T_{SP4}+\;T_{SP8}}{T_{med}^{max}\left(age\right)}\right)T_{SP4}$ are the logistic growth functions for SP4 and SP8 proliferation in the thymic medulla, respectively; $\mu_{4}T_{SP4}$ and $\mu_{8}T_{SP8}$ represent, respectively, SP4 and SP8 cell deaths; $\varphi_{4}$ and $\varphi_{8}$ are rate constants of DP-to-SP4 and DP-to-SP8 differentiation, respectively (d^-1^); $\varepsilon_{4}$ and $\varepsilon_{8}$ are rate constants of SP4 and SP8 outflows, respectively (d^-1^); $\lambda_{4}$ and $\lambda_{8}$ are rate constants of SP4 and SP8 proliferation, respectively (d^-1^); and $\mu_{4}$ and $\mu_{8}$ are SP4 and SP8 cell death rate constants, respectively (d^-1^).

The age-dependent functions $T_{cort}^{max}\left(age\right)$ and $T_{med}^{max}\left(age\right)$ were parameterized using a combination of different factors such as thymus weight loss (relatively to thymus weight in infants), the relative proportion of TES, the cortico-medullary ratio and the total thymic cell count in infants (**Figure 1B**). Linear and nonlinear forms were tested to describe the age-related changes in the thymic wet weight [ WW(age) ], the relative proportion of TES [ TES(age) ], and the cortico-medullary ratio [ CM(age) ]. A nonlinear model based on the Hill equation for WW(age) and nonlinear exponential models for TES(age) and CM(age) were finally selected, to quantitatively account for age-related changes in the respective variables.

Overall, the age-dependent functions of maximal thymocyte number in the thymic cortex [ $T_{cort}^{max}\left(age\right)$ ] and medulla [ $T_{med}^{max}\left(age\right)$ ] were described by the following equations:


(5) $$T_{cort}^{max}\left(age\right)=\;\frac{T_{0}\text{CM}\left(age\right)}{\text{CM}\left(age\right)+1}*\frac{\text{WW}\left(age\right)*\text{TES}\left(age\right)}{\text{WW}\left(0\right)*\text{TES}\left(0\right)},$$

(6) $$T_{med}^{max}\left(age\right)=\;\frac{T_{0}}{\text{CM}\left(age\right)+1}*\frac{\text{WW}\left(age\right)*\text{TES}\left(age\right)}{\text{WW}\left(0\right)*\text{TES}\left(0\right)},$$

where $WW\left(age\right)=WW_{BL}\;\left(1-\;\frac{age^{\gamma }}{age^{\gamma }+EC_{50}^{\gamma }}\right)$ represents the age-dependent thymus wet weight function; $TES\left(age\right)=\;b_{tes}*e^{-k_{tes}*age}\;$ represents the age-dependent relative proportion of TES function; $CM\left(age\right)=\;b_{cm}*e^{-k_{cm}*age}$ represents the age-dependent cortico-medullary ratio function; $T_{0}$ is the absolute total number of thymocytes in infants (0- to-1 year of age); $WW_{BL}$ stands for the thymus wet weight in infants; $EC_{50}$ is the age corresponding to the 50% of the maximum decrease in thymus wet weight; γ is a Hill coefficient; $b_{tes}$ and $b_{cm}$ are regression coefficients representing the relative proportion of TES and cortico-medullary ratio in infants; $k_{tes}$ and $k_{cm}$ are regression coefficients representing the slope of the relative proportion of TES and cortico-medullary ratio decrease functions.

### 2.3 Quantitative analysis workflow

Model analysis was performed in three sequential steps: model calibration, model validation, and simulations including sensitivity analyses. Model performance was evaluated using multiple criteria including diagnostic plots, uncertainty of parameter estimates, and values of objective function.

#### 2.3.1 Model calibration

Parameters related to thymocytes homeostasis and age-dependent thymus involution were estimated separately. The thymus involution sub-model was calibrated using the data on thymus wet weight (24), data on the relative proportion of TES [derived from total thymus, lymphatic tissue and lymphocytic perivascular space volume data in human thymus (3, 6, 34, 35)], the total number of thymocytes in infants [derived from (6) and (25)], and data on the thymic cortico-medullary ratio (10). Parameter values in Equations 5, 6 were estimated via nonlinear regression on the respective data (**Table 1**). Parameter uncertainty was assessed through a Standard Error (SE) and Relative Standard Error (RSE) calculation for each parameter in the thymus involution sub-model, with an RSE< 50% being considered as acceptable.

The thymocyte homeostasis sub-model was calibrated using derived physiological ranges of DN, DP, SP4 and SP8 cells in 0-to-1 year old infants (Section 2.1.2) and the available information on thymocyte kinetics (**Table 1**). For calibration purposes, the maximal numbers of thymocytes in the cortex ( $T_{cort}^{max}$ ) and the medulla ( $T_{med}^{max}$ ) in infants were fixed using mean predicted values from the thymus involution model for the 0-to-1 year of age group. In particular, parameter values were adjusted to reproduce the quasi steady-state turnover of DN, DP, SP4 and SP8 thymocytes within observed physiological ranges. An uncertainty analysis also considered the determination of admissible ranges of model parameters (generalized estimates), which were initially quantified via a local sensitivity analysis (model-based estimates) and further adjusted according to published experimental data (experimental estimates).

A further model analysis was performed on physiologically plausible parameter sets which satisfied the condition of quasi steady-state levels of thymocytes, within their physiological ranges (47). A Latin hypercube sampling (LHS) method with a uniform distribution was used for parameter value sampling in the thymocyte homeostasis model (Equations 1–4). Generalized estimates were used to run the LHS (**Table 2**). Calculated quasi steady-state levels of DN, DP, SP4 and SP8 cells for each of the 150,000 parameter sets were then verified to fall within their respective physiological ranges.

**Table 2:** Estimates of the calibrated parameters in the thymus involution model.

Parameter	Value	SE	RSE, %	Data
$WW_{BL}$ , g	20.652	0.994	4.8	(24)
$EC_{50}$ , y	94.729	4.285	4.5	(24)
γ	3.721	0.885	23.8	(24)
$k_{tes}$ , %*y^-1^	0.035	0.004	11.8	(3, 6, 34, 35)
$b_{tes}$ , %	93.344	5.343	5.7	(3, 6, 34, 35)
$k_{cm}$ , y^-1^	0.035	0.004	11.4	(10)
$b_{cm}$	2.77	0.206	7.4	(10)

#### 2.3.2 Sensitivity analysis

Local and global sensitivity analyses were performed to define admissible parameter ranges and to quantify how an arbitrary change in model parameters would impact thymic function; parameters were then ranked with respect to the estimated impact. A partial rank correlation coefficient (PRCC) was used as a global sensitivity characteristic (48). A global sensitivity analysis was performed by sampling parameters from physiologically plausible sets. A graphical check on data linearity and heterogeneity was performed prior to the sensitivity analysis. A statistical significance threshold was defined by introducing a “dummy” parameter in the sensitivity analysis; this parameter acted as a “negative control”, as it did not appear in the model equations and thus would not have affected the model (48).

#### 2.3.3 Model validation

Model validation was conducted for the thymus involution model, at each model development stage, using data available from literature sources (6, 25). Data on the relative thymic cortex volume $T_{cort}^{\text{max}\_relvol}$ *vs*. age were used to validate the model prediction for $T_{cort}^{max}$ (6). The validation data (thymic cortex relative volume) differed from the model output (thymic cortex cell count); thus, for validation purposes, we modified Equation 5, from describing the maximal cell number in cortex ( $T_{cort}^{max}$ ), to Equation 7 for the relative cortex volume ( $T_{cort}^{\text{max}\_relvol}$ ). Validation data and model predictions were normalized to the corresponding values for 0-to-1 year old infants, to synchronize initial conditions.

The relative thymic cortex volume ( $T_{cort}^{\text{max}\_relvol}$ ) was described by the following equation:


(7) $$T_{cort}^{max\_relvol}=\;\frac{\text{CM}\left(age\right)}{\text{CM}\left(age\right)+1}\left(\frac{\text{WW}\left(age\right)*\text{TES}\left(age\right)}{\text{Dens}\left(age\right)}\right)\left(\frac{1}{\text{Vol}\left(age\right)}\right),$$

where TES(age) is the age-dependent relative TES proportion function (see Equations 5, 6 for further details); WW(age) is the age-dependent thymus wet weight function (see Equations 5, 6 for further details); CM(age) is the age-dependent cortico-medullary ratio function (see Equations 5, 6 for further details); Dens(age) is the age-dependent thymic density (g/cm^3^) function, estimated according to thymus wet weight and volume data (6); $Dens\left(age\right)=\;k_{d}*age+b_{d}$ ; Vol(age) is the age-dependent thymic volume (cm^3^) function, estimated according to thymus volume data (6); $\;Vol\left(age\right)=\;k_{v}*age+b_{v}$ ; where $b_{d}$ and $b_{v}$ are regression coefficients representing thymic density and thymic volume in infants, and $k_{d}$ and $k_{v}$ are regression coefficients representing, respectively, the slopes of thymic density and thymic volume decrease functions.

The predictive power of the fully-developed thymocyte dynamics model was assessed via a comparison of simulations to empirical data on the relative proportions of DN, DP and SP cells and absolute numbers of total thymocytes (6, 25–33).

### 2.4 Software

Data digitization was performed using WebPlotDigitizer, version 4.6 (https://apps.automeris.io/wpd/). Extracted experimental and clinical data were gathered in the database using Microsoft Excel 365 (https://office.microsoft.com/excel). Data visualization (packages: ggplot2, cowplot, gridExtra, scales), model development (packages: dplyr, tidyr, stringr, stats, nlstools, deSolve), model simulations (RxODE package) and sensitivity analyses (packages: epiR, sensitivity) were performed in the R Statistics software, version 4.0.2 (R-project, www.r-project.org).

## 3 Results

### 3.1 Integrative model development

#### 3.1.1 Thymus involution dynamics

The range of the total number of thymocytes in 0-to-1 year old infants, $T_{0}$ , was identified as [2.2*10^10^; 5.8*10^10^] for Equations 5, 6 (see **Supplementary Table 1**). Model parameters for the thymus involution model are presented in **Table 2**.

Parameter uncertainty estimation was assessed based on the RSE and each parameter was assessed as identifiable (RSE< 50%). Goodness-of-fit plots are presented in the **Supplementary Materials** (**Supplementary Figures 1**–**3**).

#### 3.1.2 Thymocyte homeostasis turnover

Physiological ranges for DN, DP, SP4 and SP8 cells were identified as, [1.3*10^9^; 3.4*10^9^], [1.2*10^10^; 3.2*10^10^], [6.3*10^9^; 1.7*10^10^] and [2.3*10^9^; 6.0*10^9^], respectively. $T_{cort}^{max}$ and $T_{med}^{max}$ in infants were fixed to values of 2.84*10^10^ and 1.05*10^10^, respectively, based on the mean prediction of the thymus involution model for the 0-to-1 year old infant group. Parameter estimates for the thymocyte homeostasis model are presented in **Table 3**.

**Table 3:** Parameter estimates for the calibrated thymocyte homeostasis model.

Parameter	Baseline Value	Experimental estimates	Model-based estimates	Generalized estimates	Relevant Ref.
ϕ , cells*d^-1^	4.8*10^5^	[10^4^; 4.8*10^5^]	[0; 8.1*10^7^]	[10^4^; 8.1*10^7^]	(36, 40)
$\varphi_{1}$ , d^-1^	0.21	[0.18; 8.20]	[0.2066; 0.2168]	[0.2066; 0.2168]	(36, 42)
$\mu_{1}$ , d^-1^	0.056	[0.049; 0.056]	[0.053; 0.063]	[0.053; 0.063]	(5, 36)
$\mu_{2}$ , d^-1^	0.5	[0.25; 3.05]	[0.487; 0.507]	[0.487; 0.507]	(36, 42)
$\lambda_{1}$ , d^-1^	1.67	[1.67; 3.33]	[1.627; 1.693]	[1.627; 1.693]	(36)
$\lambda_{2}$ , d^-1^	3.125	[3.125; 5.988]	[3.106; 3.233]	[3.106; 3.233]	(36)
$\varphi_{4}$ , d^-1^	0.8	[0.004; 1.930]	[0.491; 1.042]	[0.491; 1.042]	(36, 42)
$\varphi_{8}$ , d^-1^	0.3	[0.002; 0.960]	[0.233; 0.505]	[0.233; 0.505]	(36, 42)
$\lambda_{4}$ , d^-1^	0.22	[0.19; 0.22]	[0; 0.877]	[0.19; 0.877]	(36, 44)
$\lambda_{8}$ , d^-1^	0.22	[0.19; 0.22]	[0; 1.338]	[0.19; 1.338]	(36, 44)
$\mu_{4}$ , d^-1^	0.005	[0; 0.06]	[0; 0.029]	[0; 0.029]	(36, 42, 44, 45)
$\mu_{8}$ , d^-1^	0.005	[0; 0.12]	[0; 0.022]	[0; 0.022]	(36, 42, 44, 45)
$\varepsilon_{4}$ , d^-1^	0.06	[0.06; 0.23]	[0.046; 0.084]	[0.046; 0.084]	(36, 44, 45)
$\varepsilon_{8}$ , d^-1^	0.06	[0.06; 0.22]	[0.036; 0.077]	[0.036; 0.077]	(36, 44, 45)

Steady-state values for the DN, DP, SP4 and SP8 thymocyte populations, with the proposed set of parameters values as specified in **Table 3**, were ~2.72*10^9^, ~2.12*10^10^, ~7.45*10^9^ and ~2.79*10^9^, respectively, and were in full agreement with experimentally observed physiological ranges for these variables. For further details, we refer the reader to the **Supplementary Materials** (**Supplementary Tables 1**, **2**).

Based on the local sensitivity analysis, lower admissible bounds for parameters ϕ , $\lambda_{4}$ , $\lambda_{8}$ , $\mu_{4}$ , and $\mu_{8}$ could not be determined and were set to zero. Final generalized estimates were identified by matching the experimental and model-based estimates. As shown in **Table 3**, all estimated parameter values were consistent with experimentally observed physiological ranges. Derivations of all experimental estimates are presented in the **Supplementary Materials** (**Supplementary Tables 3**, **4**).

### 3.2 Critical parameters

**Figure 2:**
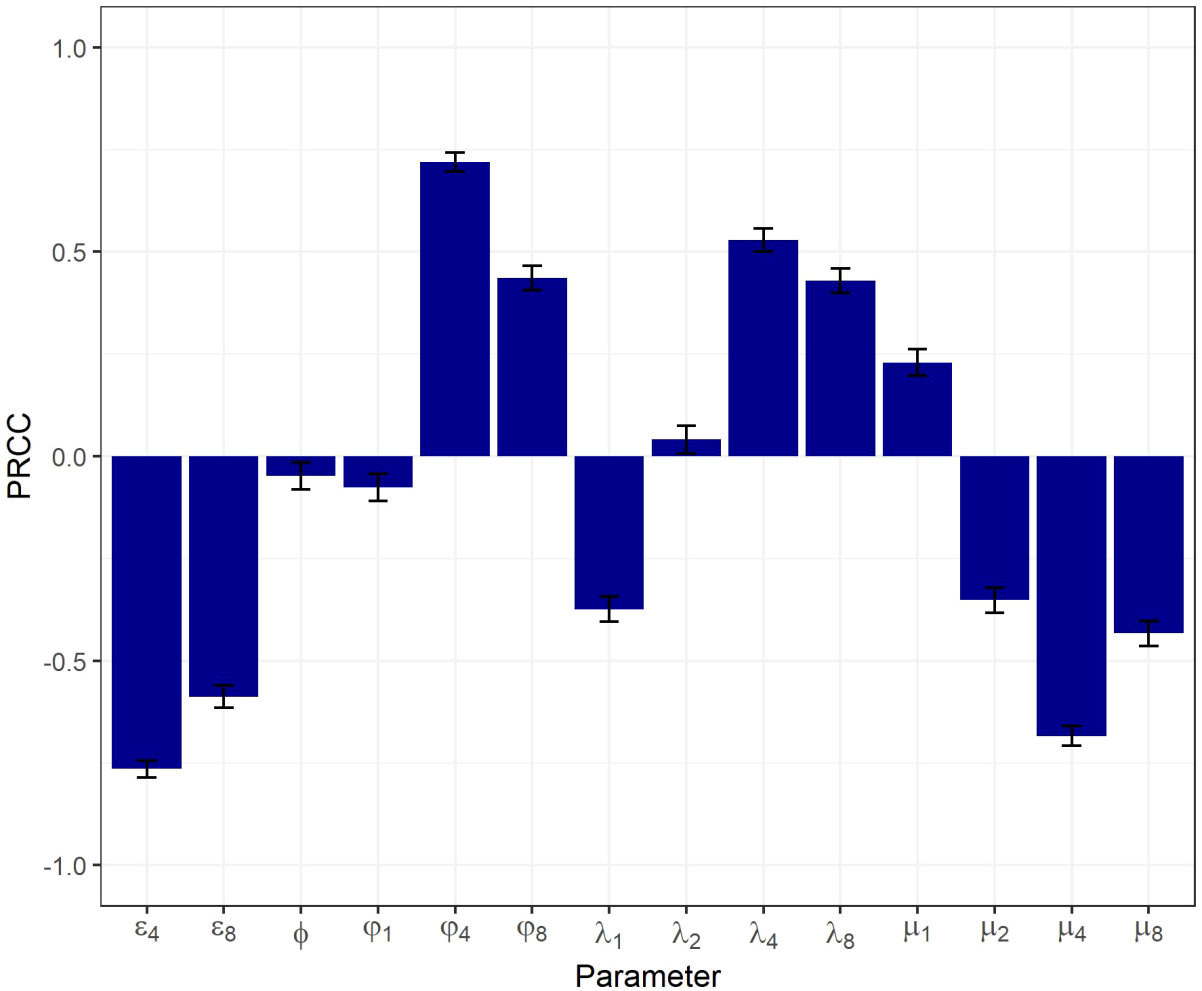
Global sensitivity analysis on a physiologically plausible set of parameters using a PRCC-based method.

The resulting physiologically plausible parameter sets upon which further analyses were performed contained 3,474 sets out of 150,000 sets generated. Results from the global sensitivity analysis are presented in **Figure 2**. The SP thymocyte number was chosen as a key output of the model, since it reflects most closely overall thymic function. Relationships between the model output and each parameter were of a monotonic, linear nature (see **Supplementary Figure 4**).

All model parameters significantly affected the output, according to the PRCC-based sensitivity analysis. The highest PRCC coefficients were predicted for $\varepsilon_{4}$ , $\varepsilon_{8}$ , $\varphi_{4}$ , $\lambda_{4}$ and $\mu_{4}$ (PRCC > 0.5), indicative of these parameters being most critical in driving the system’s behavior.

### 3.3 Model performance evaluation

**Figure 3:**
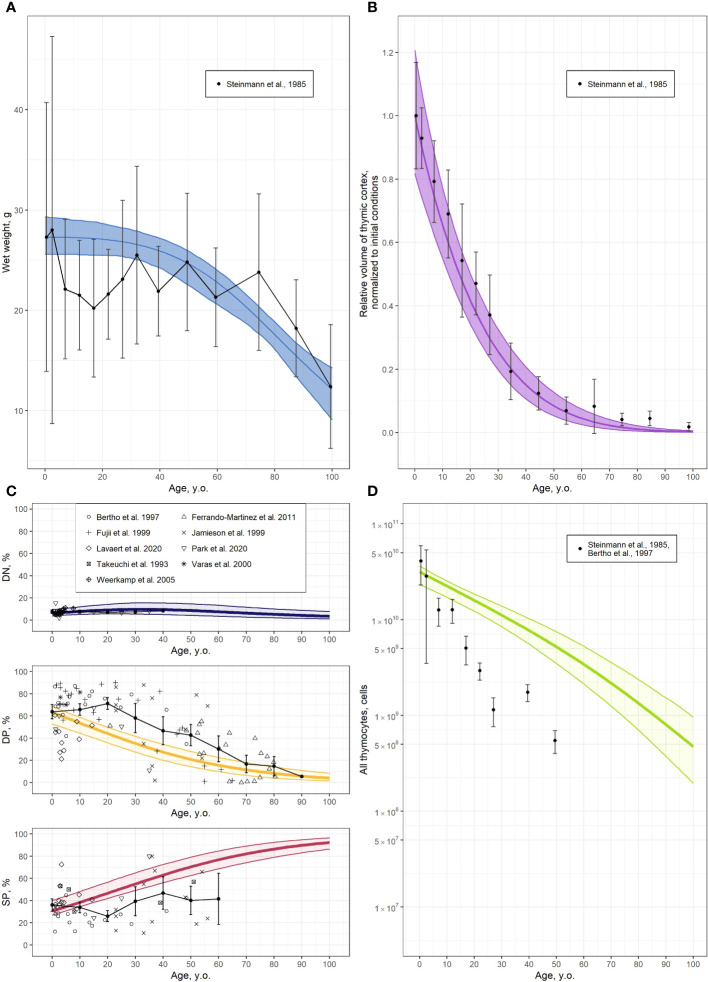
Validation of the thymocyte dynamics model. **(A)** Thymus wet weight [solid line and shaded area represents the model predicted mean with 95% CI uncertainty band, respectively, and symbols and error bars stand for the mean and 95% CI of clinical data (6)]; **(B)** Relative volume of thymic cortex, normalized to initial value, derived from the modified function in Equation 7 [solid line and shaded area represents the model predicted mean with 95% CI uncertainty band, respectively, and symbols and error bars stand for the mean and 95% CI of clinical data (6)]; **(C)** Relative proportions of DN, DP and SP cells [solid line and shaded area represents the model predicted mean with 95% CI uncertainty band, respectively, symbols stand for clinical data (25–33) and black solid line with error bars represents moving average with 95% CI (10 years step)]; **(D)** Absolute values of total thymocyte numbers [solid line and shaded area represents the model predicted mean with 95% CI uncertainty band, respectively, and symbols and error bars stand for mean and 95% CI of clinical data derived from (25, 6); see also **Supplementary Table 1**].

Model validation results are presented in **Figure 3**. The band represents the 95% uncertainty of each model-predicted solution. The function capturing the age-dependent decrease in thymus wet weight, as a part of the thymus involution model, was successfully validated based on published thymus weight data (6) (**Figure 3A**). Due to differences in baseline values (0-to-1 year of age group) between calibration and validation datasets, the thymus wet weight model was adjusted from the validation data to a baseline value of 27.3 g. Validation results of the composite model outcome ( $T_{cort}^{max}$ ) after adjustment (see Section 2.3.3. for further details) are shown in **Figure 3B**. Estimates of the calibrated parameters and goodness-of-fit plots for thymic density ( Dens(age) ) and thymic volume ( Vol(age) ), which are needed for validation of the thymus involution model (Equation 7), are presented in the **Supplementary Materials** (**Supplementary Table 5**; **Supplementary Figures 5**, **6**).

Combining the thymocyte homeostasis sub-model with the thymus involution sub-model provided the overall integrative model for describing thymocyte dynamics with age. The thymocyte dynamics model was validated by comparing its output to the absolute values and relative fractions of thymocyte sub-types curated from various sources, as presented in **Figures 3C, D**. A moving average with a 10-year step was applied to the relative proportions of thymocyte sub-type data, to visualize data variability and compare such *vs*. model predictions.

According to these validation results, the integrative model adequately described the age-related thymic function, both in qualitative and quantitative terms. Relative proportions of DN and DP thymocytes decreased with age, while the proportion of SP cells increased (**Figure 3C**). Such a process can be explained due to a faster involution of the cortex *vs*. the medulla. Inconsistencies arose in the predictions of DP and SP counts in the 10- to 30-year age group and the >50-year age group for the SP cell population, primarily due to the sparsity and relative lack of data for these groups (**Figure 3C**). The absence of data on the absolute values of total thymocyte numbers in human, combined with the necessity to merge data from various sources to calculate the absolute number of thymocytes, resulted in a partial discrepancy between model predictions and the data (**Figure 3D**). Nevertheless, the overall model adequately reproduced the age-dependent trends in both total thymocyte counts and all thymocyte sub-population counts.

### 3.4 Prediction of thymic function for clinically relevant settings

**Figure 4:**
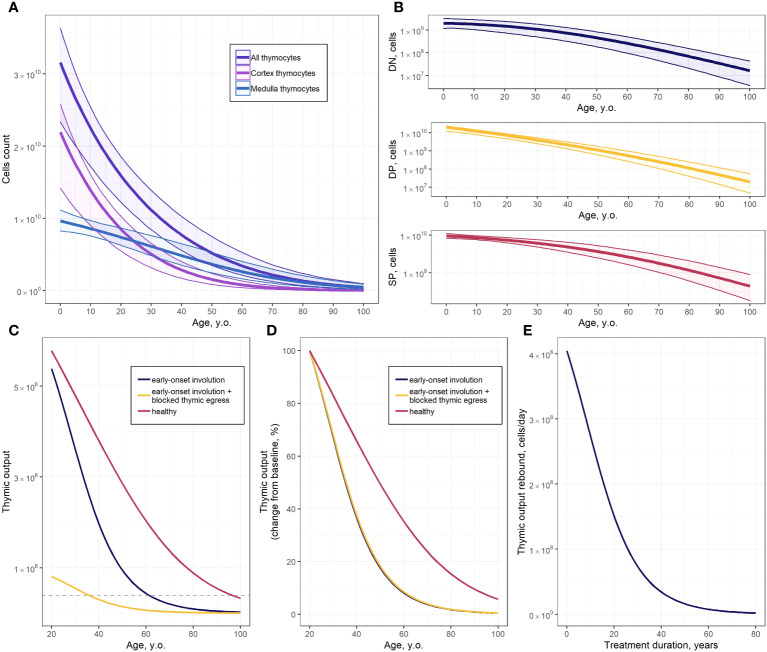
Predictions of the thymocyte dynamics model. **(A)** Absolute values of all thymocyte counts and of cortex and medulla thymocytes (solid line and shaded area represents the model predicted mean with 95% CI uncertainty band, respectively); **(B)** Absolute values of DN, DP and SP thymocyte populations (solid line and shaded area represents the model predicted mean with 95% CI uncertainty band, respectively); **(C)** Predicted mean of thymic output for healthy subjects, subjects with early-onset thymus involution, and subjects with early-onset thymus involution and blocked thymocytes egress (dashed line – thymic output threshold for fingolimod discontinuation); **(D)** Predicted mean of SP cell counts, expressed as changes from baseline, for healthy subjects, subjects with early-onset thymus involution, and subjects with early-onset thymus involution and blocked thymocytes egress; **(E)** Predicted thymic output rebound after thymocyte egress restoration in 20-year old subjects with early-onset thymus involution.

The integrated model of thymocyte dynamics was next used to perform simulations of thymus function changes with age (**Figure 4**), specifically, to predict thymocyte counts in the different anatomical compartments: the cortex, the medulla, and the overall thymus (**Figure 4A**); and of the various cell populations, including DN, DP, and SP cells (**Figure 4B**). The model explicitly captured the dynamics of main thymocyte populations (DN, DP, SP4 and SP8) and implicitly included various sub-populations, such as thymic-derived regulatory T-cells (tTreg) and re-entered mature lymphocytes. Given that tTreg cells constitute ∼1 – 3% of SP4 cells in the thymus, it becomes feasible to predict the absolute number of these cells (49, 50). In infants, the calculated tTreg count was 3.2*10^6^ – 9.6*10^6^ cells per gram of thymus, a value which roughly aligns with experimental data (14.1 ± 4.2*10^6^ cells per gram of thymus (51)).

Thymic function simulations were performed for subjects under three sets of physiological conditions: healthy subjects, subjects with an early onset of thymus involution, and subjects with an early onset of thymus involution and with blocked thymocyte egress (**Figures 4C, D**). Early-onset thymus involution may occur under certain pathological conditions, while thymocyte egress blockage may occur under therapeutic pharmacological interventions with certain immunomodulators such as fingolimod. The corresponding model predictions were expressed as the rate of exported SP cells (cells*d^-1^). Average parameter values from physiologically plausible parameter sets were used to simulate healthy subjects. Early-onset thymus involution was modeled by reducing the $EC_{50}$ parameter value to $EC_{50}$ = 40 years. Thymocyte egress blockage was modeled by lowering the egress rates of SP4 ( $\varepsilon_{4}$ ) and SP8 ( $\varepsilon_{8}$ ) cells from 0.076 d^-1^ and 0.082 d^-1^ to 0.011 d^-1^ and 0.012 d^-1^, respectively. A detailed description of the parameter values used to simulate both early-onset thymus involution and thymocyte egress blockage scenarios is provided in the **Supplementary Materials** (**Supplementary Table 6**) (52).

One of the clinical factors limiting the use of fingolimod is the development of severe lymphopenia. According to European Medicines Agency (EMA) guidelines, fingolimod-based treatment should be discontinued when the absolute lymphocyte count in blood drops below 200 cells/mm^3^ (53, 54). The average duration of fingolimod treatment prior to developing severe lymphopenia was identified through simulations of the impact of fingolimod discontinuation on thymic output. A detailed description of the estimation of the average threshold of thymic output for fingolimod therapy discontinuation is presented in the **Supplementary Materials** (**Supplementary Table 7**) (41, 55–57).

We determined a model-based, ∼1.1-fold difference in absolute numbers of exported SP cells, when comparing healthy subjects *vs*. subjects with early-onset thymus involution in a 20 year-of-age group (**Figure 4C**); this difference in thymic count, however, increased with age (**Figure 4D**). Blockage of thymocyte egress led to a decrease in the absolute values of exported SP cell counts (**Figure 4C**). However, no significant differences in the rate of thymus involution were observed, with respect to subjects’ age, with or without thymocyte egress blockage (**Figure 4D**, blue and yellow lines). Considering the calculated thymic output threshold for fingolimod discontinuation, grade 4 lymphopenia is expected to develop after ∼19 years of continuous fingolimod, with drug treatment initiated at the age of 20 (**Figure 4C**). The effect of fingolimod treatment duration on the thymic output rebound after drug discontinuation in 20-year old multiple sclerosis patients is presented in **Figure 4E**; the shorter the treatment duration, the greater the extent of thymocyte rebound after fingolimod discontinuation (**Figure 4E**). However, this would also present a higher risk of severe lymphopenia development (**Figure 4C**).

## 4 Discussion

The development and validation of an integrative mechanistic model of thymocyte dynamics is a challenging task, given not only the inherent complexity of the underlying biology and physiology, but also due to intrinsic and extrinsic uncertainty and variability in the data, as can be seen in **Figure 3**. These challenges are further compounded by the lack of longitudinal thymocyte data in humans. Framing thymocyte homeostasis while identifying and extracting consistency across multi-source, multi-scale experimental data and biological knowledge is an essential and demanding task for the development of a quantitative, dynamic, physiologically-based mathematical model. This type of models may then be used to perform predictive simulations under scenarios of various pathophysiological conditions and/or therapeutic pharmacological interventions. The model of thymic function we proposed here is consistent with current qualitative and quantitative knowledge on homeostasis of various thymocyte populations and on age-related differences in thymic function. Furthermore, the model adequately described the changes in thymic function under certain pathological conditions, such as multiple sclerosis, and pharmacological immunomodulatory intervention, such as fingolimod administration. Finally, our model-based analysis revealed parameters ( $\varepsilon_{4}$ , $\varepsilon_{8}$ , $\varphi_{4}$ , $\lambda_{4}$ and $\mu_{4}$ ) which are most critical in determining the system performance characteristics, which may be “targeted” to improve thymic function, e.g., under pharmacological modulation.

Model development and validation were based on a broad set of published experimental and clinical data, on thymocyte homeostasis and age-dependent thymus involution. Our model calibration approach is more exhaustive than previous thymus modeling efforts, whereby single sets of mainly murine data [thymocyte kinetic data in normal thymus (16) and in thymus with induced T-cell development abnormalities (12, 13); steady-state thymocyte counts (14)] were used for model development. Despite the heterogeneity of data sources, calibration of parameter estimates in the model resulted in a close agreement with previously published models (**Supplementary Table 8**). However, the inflow rate of thymocyte precursors was difficult to compare across existing mathematical models of the thymus, given that cell content within the DN compartment varied among models. Differences in underlying structures between the presently described *vs*. previously published models resulted in differences for several parameter estimates. Because not every published model incorporates DP cell proliferation rate or the control of cell transition from the cortex to the medulla, estimates for the DP proliferation rate and the DP-to-SP4 and DP-to-SP8 differentiation rates were approximately an order of magnitude higher than respective parameter estimates from previously published models (**Supplementary Table 8**).

The quality of the data used for model verification along with the utilization of mouse data may influence the reliability of the model predictions. All quantitative data identified through our systematic literature review were used for parameter estimation and model validation. No assessment was made regarding publication bias in the experimental and clinical data, and there remains a need for establishing quantitative criteria to distinguish high quality experimental immunological data from lesser quality ones. Addressing this issue involves the consideration of options proposed by the QSP community, such as focusing on parameter variability and model validation (58). Several dynamic characteristics of various thymocyte subsets were extracted from experiments conducted in mouse (**Supplementary Table 3**). The derivation of experimental estimates for model parameters (**Table 3**) involved scaling from mouse to human (36, 40), except for parameters $\mu_{1}$ , $\lambda_{1}$ , $\lambda_{2}$ , $\lambda_{4}$ , $\lambda_{8}$ , $\varepsilon_{4}$ and $\varepsilon_{8}$ (**Supplementary Table 4**). According to the sensitivity analysis results (**Figure 2**), parameters $\varepsilon_{4}$ and $\varepsilon_{8}$ exerted the most significant influence on thymic function. Discrepancies in the derivation of these parameters could potentially impact model performance. However, the consistency of model estimates with experimental values (**Table 3**), as well as the ability of the model to adequately describe human data (**Figures 3C, D**) provided support in using such data. Nevertheless, model verification should be refined as more human data become available.

Estimates of maximal thymocyte numbers in the cortex and the medulla were derived, both to quantify age-related thymus involution and to limit excessive, non-physiological cell proliferation and cell flow across thymus compartments. A similar approach to thymocyte growth control has been implemented in the Ye et al. model, whereby a time-dependent function of the thymus epithelial space was used to limit the proliferation of each subset of thymocytes (15). We elected to consider cortex and medulla in the thymus separately, since involution rates in these areas significantly differ from each other (10). The derived quasi steady-state values of each thymocyte subset were lower than the estimated maximal number of thymocytes in the cortex and the medulla, which provided an opportunity to allow for an increase in cell proliferation as may occur under infection or specific therapeutic intervention conditions.

The sensitivity analysis showed that the egress, differentiation, proliferation and death rates of SP (especially SP4) cells were the most critical system parameters controlling thymic function. Sensitivity analysis results were consistent with those predicted by other mathematical models, whereby the SP4 emigration rate, together with T-cell division and death rates were found to be the most important parameters affecting the concentration of TRECs and, subsequently, thymic output (15). Moreover, the sensitivity analysis revealed a low impact of thymocyte precursor inflow on thymic output, which can be explained by a limited number of TSP niches in the thymic cortex (36). While age-dependent involution is characterized by a stronger loss of cortex as compared to the medulla, parameters most critical for thymic function were those affecting SP cells, as a more rapid depletion of DP and DN cells resulted in a conditional accumulation of SP cells. The observed inconsistencies of model prediction with experimental data, particularly regarding the relative number of SP cells, can be attributed to the absence of processes in the model required to mechanistically describe other immune cell differentiation (e.g., tTreg) and the process of mature lymphocyte re-entering.

The developed model was used to simulate several clinically relevant scenarios reflective of patho-physiological conditions and specific pharmacological interventions. For example, a condition of early-onset thymus involution, through a setting of low levels of SP cells in the thymus could be implemented in the mechanistic model; such a condition may be linked to chronic autoimmune conditions, e.g. multiple sclerosis. Patients with multiple sclerosis exhibit early-onset of thymus involution, since they feature levels of TREC-containing CD4+ and CD8+ T cells which would be equivalent to those found in 30-year old healthy subjects (59). Another clinically relevant scenario which may be prospectively simulated with the present model relates to chronic treatments using pharmacological immunomodulators such as fingolimod, a small molecule drug which inhibits S1P receptors (60). S1P/S1PR1 signaling is indeed required for thymocytes to emigrate out of the thymus (61). S1P activation of S1PR1 on mature SP cells may allow thymocytes to exit the thymus and enter the circulation. Accordingly, a lack of S1PR1 would result in thymocytes being unable to leave the thymus (61). It has been shown that thymocyte egress is delayed upon fingolimod administration (60, 62).

The performed simulations provided a mechanistic basis for understanding thymic output dynamics and its dependence on age, in cases of healthy subjects, multiple sclerosis patients, and patients on fingolimod treatment. Our predictive simulations showed that thymic output significantly differed between healthy subjects and multiple sclerosis patients, even though these systemic interventions would not affect the slope (rate) of the thymus involution process. One may infer from such simulations results that, upon fingolimod discontinuation, the probability of multiple sclerosis relapse would decrease with age, since thymic output is significantly lower at more advanced ages. Such an inference is actually supported by clinical evidence, whereby patients at younger ages exhibited a higher risk of relapse following fingolimod discontinuation (63, 64). However, it is also important to note that relapse would not occur solely due to a rapid re-entry of lymphocytes into the periphery and the central nervous system; relapse has been observed even under conditions whereby lymphocyte counts remained depressed (65). It is also important to understand the effect of fingolimod treatment duration on the risk of developing severe lymphopenia (54). Our model predictions provided insights into how the duration of fingolimod use may affect thymus function. An average treatment duration of ∼19 years in 20-year old subjects, as defined by the present model, was qualitatively consistent with reports of infections (e.g., cryptococcal meningitis) which may occur after 2-3 years of treatment (54). Indeed, thymus function varies among patients, depending on their individual characteristics, and our simulation results reflected average trends. The next challenge lies in individualizing model-based simulations, based on a subject’s drug pharmacokinetics and transcriptomic and immunophenotypic data.

The proposed mechanistic model of thymocyte dynamics provides a consistent description of T-cell development, in agreement with existing clinical data – yet it also comes with a number of limitations. For example, we used a deterministic approach to quantify and extrapolate thymic function dynamics, in relation to time and age. Therefore, in order to follow the parsimony principle and to avoid overt parameter identifiability issues (66), we simplified the model structure. Specifically, we did not take into account all aspects and all intermediate stages of T-cell development. Another potential limitation is that our model did not consider the development of other immune cells that takes place in the thymus, such as tTreg, and mature re-entering lymphocytes. Although the model allows for the calculation of tTreg numbers in the thymus as a small (1 – 3%) fraction of SP4 cells (50), one of the model limitations is related to the inability to mechanistically describe tTreg development and, consequently, assess the velocity of this process, explore other paths to tTreg differentiation [such as evaluating the contribution of an alternative CD25- progenitor Treg subpopulation (49)], or investigate the impact of autoimmune diseases such as Myasthenia gravis (67). Accounting for the re-entry of mature lymphocytes may represent a priority option for model expansion (35). To address this, the model should incorporate at least one population of peripheral lymphocytes. Moreover, since the ability to re-enter differs between naïve and previously activated T-cells (68), incorporating a dynamic description of peripheral T-cell behavior could be beneficial. Capturing re-entered lymphocytes into distinct subpopulations and providing a more detailed description of the latter stages of thymocyte development may well enhance the model’s accuracy in quantitative predictions and overall performance. However, this would necessitate a more extensive dataset comprising experimental and quantitative data on corresponding cellular dynamics and remains to be systematically addressed in the future.

Nevertheless, the mechanistic model featured here may be used as a basis for further multi-scale modeling of T-cell dynamics. The current version of the model may also be extended to capture scenarios of infections (e.g., HIV) and/or drug- or stress-induced thymus atrophy processes. Such model extensions will provide a tool for further quantitative and mechanistic understanding of thymus function, under various conditions and combinations of homeostatic disruptions.

## 5 Conclusions

An integrative mechanistic model of thymocyte dynamics in human was developed and validated using multi-scale experimental and clinical data. The model integrated key mechanistic processes of human thymopoiesis, including homeostasis of various thymocyte populations and age-related changes in thymic function. A model-based sensitivity analysis revealed that the SP cell export, differentiation, proliferation and death were the more important processes influencing overall thymic function. Our model-based simulations suggested that the clinically observed decrease in relapse risk with age, in multiple sclerosis patients who would have discontinued fingolimod therapy, can be explained mechanistically by decreased thymic output with age. A quantitative assessment of the relationship between thymic output and duration of fingolimod treatment revealed an average therapy duration of ∼19 years until the development of grade 4 lymphopenia. The model described here may be interrogated to quantitatively explore various scenarios of pharmacological interventions and personalized treatments relevant to drug development. It may also serve as a basis for further mechanistic extensions, including consideration of additional T-cell dynamics regulation processes as well as the impact of infections and drug- or stress-induced thymus atrophy scenarios.

## Data availability statement

The original contributions presented in the study are included in the article/**Supplementary Material**. Further inquiries can be directed to the corresponding author.

## Author contributions

VK: Conceptualization, Data curation, Formal analysis, Investigation, Methodology, Validation, Visualization, Writing – original draft, Writing – review & editing. KP: Conceptualization, Methodology, Validation, Funding acquisition, Supervision, Writing – review & editing. GH: Conceptualization, Methodology, Validation, Writing – review & editing. GB: Conceptualization, Methodology, Validation, Supervision, Writing – review & editing.
