# Supplementary material for: An integrative mechanistic model of thymocyte dynamics
Source: Front Immunol. 2024 Feb 26;15:1321309. doi: 10.3389/fimmu.2024.1321309 (PMC10925769; doi:10.3389/fimmu.2024.1321309)
Supplement: Supplementary file 1 [file DataSheet_1.docx]

Supplementary Material

An integrative mechanistic model of thymocyte dynamics

Victoria Kulesh^1,2*^, Kirill Peskov^1,2,3,4^, Gabriel Helmlinger^5^, Gennady Bocharov^2,6,7^

*** Correspondence:** Victoria Kulesh: [viktoriaan37@gmail.com](mailto:viktoriaan37@gmail.com)

# Supplementary Figures and Tables

## Supplementary Tables

Supplementary Table 1. Derivation of total thymocyte counts

|  | - **Wet weight, g** (1) | - **Thymocytes, cells/g** (2) | - **Thymocytes, cells** [*weight, g * thymocytes, cells/g*] |
| --- | --- | --- | --- |
| **0-to-1 year old infants** | | | |
| - mean | - 27.3 | - 1.5*10^9^ | - 4*10^10^* |
| - Standard deviation (sd) | - 16.4 | - 0.4*10^9^ | - 2.77*10^10^** |
| - Standard error (se) | - 6.532 | - 0.133*10^9^ | - 9.23*10^9^*** |
| - lower limit 95%CI | - 14.18 | - 1.24*10^9^ | - **2.2*10^10^****** |
| - upper limit 95% CI | - 40.42 | - 1.76*10^9^ | - **5.8*10^10^****** |

Blue-colored values – literature data;

Red-colored values – derived data;

Values **in bold** – selected range of total numbers of thymocytes among infants

*derived by multiplying wet weight (g) measure by thymocyte (cells/g) measure

**derived using a formula for variance of a product of two independent random variables ($Var\left( XY \right)=Var\left( X \right)*Var\left( Y \right)+Var\left( X \right)*\left( E\left( Y \right) \right)^{2}+Var\left( Y \right)*\left( E\left( X \right) \right)^{2}$)

***derived using the formula $se= \frac{sd}{\sqrt{n}}$, where n – number of subjects in the 0-to-1 year of age group (2)

****derived using the confidence interval formula $CI=mean \pm Z_{\frac{\alpha}{2}}*se$

The same approach was used to derive thymocyte counts in other age groups (then used for model validation).

Supplementary Table 2. Physiological ranges of DN, DP, SP, SP4 and SP8 thymocyte populations

|  | **DN cells**  (n = 45) | **DP cells**  (n = 52) | **SP cells**  (n = 33) | **SP4 cells**  (n = 30) | **SP8 cells**  (n = 30) |
| --- | --- | --- | --- | --- | --- |
| Average percentage of thymocytes in the “0-to-1 year of age” group (2–8) | 5.9 % | 55.7 % | 38.9 % | 28.5 % | 10.4 % |
| Total thymocyte count (*see Table S1 for derivation*) | [2.2*10^10^; 5.8*10^10^] | | | | |
| Thymocyte count [*total thymocyte count upper/lower limit * median percentage of thymocytes in thymus*] | **[1.3*10^9^; 3.4*10^9^]** | **[1.2*10^10^; 3.2*10^10^]** | **[8.6*10^9^; 2.3*10^10^]** | **[6.3*10^9^; 1.7*10^10^]** | **[2.3*10^9^; 6.0*10^9^]** |

Values **in bold** – selected physiological ranges for each thymocyte subset.

Supplementary Table 3. Dynamic characteristics of different thymocyte subsets (literature and model data)

| **Thymocyte subset** | **Number of cells** | **Residence time** | **Turnover rate** | **Production rate** | **Export rate** |
| --- | --- | --- | --- | --- | --- |
| TSP | 10 niches for cells (mouse data, (9,10)  160 cells (mice data, (9)) | Experimental data:  2.5 days (mouse data, (9)) |  |  |  |
| ETP (DN1) | 2-3 * 10^4^ cells (mouse data, (9)) | Experimental data:  9 – 12 days (mouse data, (9)) |  |  |  |
| DN2 | 2-3 * 10^4^ cells (mouse data, (9)) | Experimental data:  2.00 – 2.75 days (mouse data, (9)) |  |  |  |
| DN3 | 2-3 * 10^6^ cells (mouse data, (9)) | Experimental data:  2 – 4 days (mouse data, (9)) |  |  |  |
| DN3a | 1.6 * 10^6^ cells (mouse data, (9)) |  |  |  |  |
| DN3b | 4 * 10^5^ cells (mouse data, (9)) |  |  |  |  |
| **Turn from DN1 to DN3 (DN1 => DN3)** |  | Experimental data:  without TSP stage:  min = 13 days;  max = 19 days;  median = 16 days;  with TSP stage:  min = 15.5 days;  max = 21.5 days; median = 18.5 days; |  |  |  |
| DN4 |  | Experimental data:  3 days (11) |  |  |  |
| **Cell turning, DN1 to DN4** |  | Experimental data:  without TSP stage:  min = 16 days;  max = 22 days;  median = 19 days  with TSP stage:  min = 18.5 days;  max = 24.5 days;  median = 21.5 days  Model data:  17.6 days (*Thomas-Va*slin et al. model (12)) | 6.5*10^5^ cells/day (mouse (13))  (turnover time = 3 days) |  |  |
| DP | 96.5 * 10^6^ cells (mouse data, (9)) | Experimental data:  3.167 days (mouse data, (9))  3.5 days (mouse data, (14))  3 – 4 days (15)  Model data:  3.17 – (*Sawicka et al.* model, based on hypothesis (16))  3.9 days (*Thomas-Vaslin et al.* model (12))  5.18 days (*Moleriu et al.* model (17))  8 days (*Sinclair et al.* model (18)) | 2.3*10^7^ cells/day (mouse data, (13)) |  |  |
| DP pre-selection | 88 * 10^6^ cells (mouse data, (9)) | Experimental data:  2.5 days (mouse data, (9))  Model data:  1.3 days (*Sinclair et al.* model (18)) |  |  |  |
| DP post-selection | 8.5 * 10^6^ cells (mouse data, (9)) | Experimental data:  0.667 days (mouse data, (9))  Model data:  4.5 days (*Sinclair et al.* model (18)) |  |  |  |
| SP | 17 * 10^6^ cells (mouse data, (9)) | Experimental data:  5.4 days (mouse data, (9)) |  |  | ~ 1 % of all thymocytes, 1-3*10^6^ cells/day (mouse data, (15)) |
| SP4 | 12 * 10^6^ cells (mouse data, (9)) | Experimental data:  5.4 days (mouse data, (9))  4.4 days (mouse data, (19))  Model data:  5.74 days (*Thomas-Vaslin et al.* model (12,20))  5.26 days (*Sinclair et al.* model (18,20))  6.85 days (*Moleriu et al.* model (17,20)) |  | 4.5*10^5^ cells/day (mouse data, (13)) |  |
| SP8 | 4 * 10^6^ cells (mouse data, (9)) | Experimental data:  5.4 days (mouse data, (9))  4.6 days (mouse data, (19))  Model data:  4.7 days (*Thomas-Vaslin et al.* model (12,20))  3.85 days (*Sinclair et al.* model (18,20))  3.11 days (*Moleriu et al.* model (17,20)) |  | 2.5*10^5^ cells/day (mouse data, (13)) |  |

Supplementary Table 4. Procedures used to transform experimental data estimates to model parameter estimates.

| **Process name** | **Detailed description of derivation** |
| --- | --- |
| **DN thymocytes** | |
| Percent of DN cells dying at β-selection stage | β-selection takes place at the DN3 stage–  70% of DN3 thymocytes die at the β-selection checkpoint (21)  $N \left( DN cells died at \beta-selection \right)$ = $N \left( DN3a \right)- N (DN3b)$ = 1.6*10^6^ – 4*10^5^ = 1.2*10^6^ cells   - $\% \left( DN cells died at \beta-selection \right)= \frac{N \left( DN cells died at \beta-selection \right)}{N (DN)}= \frac{1.2*{10}^{6}}{3*{10}^{6}}$ = 40% of DN thymocytes dying at the β-selection stage |
| DN cell lifetime | $DN cells \left\{ \begin{aligned} 40\% of DN cells turn from DN1 to DN3 \\ (as 40\% of DN thymocytes die at \beta-selection stage) \\ 60\% of DN cells turn from DN1 to DN4 \end{aligned} \right.$  DN cell lifetime = 60% * DN (all) residence time (median) + 40% * (DN1 => DN3) residence time (median)  without TSP stage:  DN cell lifetime = 0.6 * 19 + 0.4 * 16 = 17.8 days  with TSP stage:  DN cell lifetime = 0.6 * 21.5 + 0.4 * 18.5 = 20.3 days |
| DN cells death rate  ($\mu_{1}$, d^-1^: [0.049; 0.056]) | $Death rate= \frac{1}{residence time}$  without TSP stage:  DN cell death rate = 1 / 17.8 = 0.056 $\mathrm{da}y^{-1}$  with TSP stage:  DN cell death rate = 1 / 20.3 = 0.049 $\mathrm{da}y^{-1}$ |
| DN cells differentiation rate  ($\varphi_{1}$, d^-1^: [0.18; 8.20]) | $Turnover rate of cells=\left( diff. rate+death rate \right)* steady-state value of cells$  $diff. rate= \frac{Turnover rate of cells}{steady-state value of cells}- death rate$  1^st^ option (mouse data):  Turnover rate data (13) –5-6 week old mice (~38 days)  Steady-state values (9) – 8-10 week old mice (~63 days)  5-6 week old mice; steady-state value adjustment: formula from (22,23):  $thymocyte count at x days age =$  $thymocyte count at birth* e^{- thymocyte loss per day *mouse age in days}$  $thymocyte loss per day$ = 0.0041 $\mathrm{da}y^{-1}$ (22,23)  $X_{8-10 w.o.}= X_{birth}* e^{-0.0041*63}$  $X_{5-6 w.o.}= X_{birth}* e^{-0.0041*38}= X_{8-10 w.o.}* e^{0.0041*25}=X_{8-10 w.o.}* e^{0.1025}\approx1.1*X_{8-10 w.o.}$  ${DNss}_{5-6 w.o.}=2.5*{10}^{6}*1.1=2.75*{10}^{6}$ cells  $diff. rate+death rate= \frac{DN turnover rate}{{DNss}_{5-6 w.o.}}= \frac{6.5*{10}^{5}}{2.75*{10}^{6}}=0.236 \mathrm{da}y^{-1}$  without TSP stage:  $diff. rate= 0.236-death rate= 0.236-0.056= 0.18 \mathrm{da}y^{-1}$  with TSP stage:  $diff. rate= 0.236-death rate= 0.236-0.049= 0.187 \mathrm{da}y^{-1}$  2^nd^ option (mouse data scaled to human):  $diff. rate+death rate= \frac{Turnover rate (scaled for human)}{DN steady-state (human)}$  $DN steady-state (human)$ = 2.36*10^9^ (mean value for 0-to-1 year of age group (range [1.3*10^9^; 3.4*10^9^] (*see Table S2*)))  *Scaling factor: 10^3^* (9,24):  $diff. rate+death rate= \frac{6.5*{10}^{6}*{10}^{3}}{2.36*{10}^{9}}=2.75 \mathrm{da}y^{-1}$  *Scaling factor: 3 * 10^3^* (9,24):  $diff. rate+death rate= \frac{6.5*{10}^{6}*{3*10}^{3}}{2.36*{10}^{9}}=8.26 \mathrm{da}y^{-1}$  Range for differentiation rate: $diff. rate+death rate \left( range \left[ 2.75 day^{-1}; 8.26 day^{-1} \right] \right)-death rate \left( range \left[ 0.049 day^{-1}; 0.056 day^{-1} \right] \right)$: [2.701 $\mathrm{da}y^{-1}$; 8.204 $\mathrm{da}y^{-1}$] |
| Number of divisions and time for 1 division, DN cells | $N_{step2}= N_{step1}* 2^{n_{divisions}}$  $1 division time= \frac{residence time}{n_{divisions}}$  According to cell numbers (*see Table S4*), the highest increase in cell number in the DN compartment occurs at the DN2 => DN3 stage:  $n_{divisions}= {log}_{2}\left( \frac{N\left( DN3 \right)}{N\left( DN2 \right)} \right)= {log}_{2}100=6.64$  Residence time in DN3 = 2 – 4 days (mean = 3 days) (*see Table S4*)  Time required for 1 division:  *Lower limit of residence time:* 2 / 6.64 = 0.30 days (1 division – 0.30 days)  *Mean value of residence time:* 3 / 6.64 = 0.45 days (1 division – 0.45 days)  *Upper limit of residence time:* 4 / 6.64 = 0.60 days (1 division – 0.60 days) |
| DN cells proliferation rate  ($\lambda_{1}$, d^-1^: [1.67; 3.33]) | $proliferation rate=\frac{1}{1 division time}$  *Lower limit of residence time:* 1 / 0.30 = 3.33 $\mathrm{da}y^{-1}$  *Mean value of residence time:* 1 / 0.45 = 2.22 $\mathrm{da}y^{-1}$  *Upper limit of residence time:* 1 / 0.60 = 1.67 $\mathrm{da}y^{-1}$ |
| Inflow of DN cells  ($\phi$, cells*d^-1^: [10^4^; 4.8*10^5^]) | According to (9,10) data (see Table S4), TSP cell count = [10 cells;160 cells]  *Scaling factor: 10^3^* (9,24): [10^4^ $cells*\mathrm{da}y^{-1}$;1.6*10^5^$cells*day^{-1}$]  *Scaling factor: 3*10^3^* (9,24): [3*10^4^$cells*day^{-1}$;4.8*10^5^ $cells*\mathrm{da}y^{-1}$] |
| **DP thymocytes** | |
| DP cell death rate  ($\mu_{2}$, d^-1^: [0.25; 3.05]) | 1^st^ option:  $Death rate= \frac{1}{residence time}$  DP residence time ≈ 4 days  death rate = 1 / 4 = 0.25 $\mathrm{da}y^{-1}$  2^nd^ option:  $y=be^{-kt}$  where $y$ – DP cell number; $t$ – time (days); $b$, $k$ – equation coefficients  Assuming an exponential decline in DP cells due to negative and positive selection, from 88 * 10^6^ cells (pre-selection DP, *see Table S4*, (9)) to 8.5*10^6^ (post-selection DP, *see Table S4*, (9)) over 2.5 days (pre-selection DP residence time, *see Table S4*, (9))), the death rate (coefficient $k$) is equal to 0.92 $day^{-1}$  3^rd^ option:  $death rate=\left( diff.rate+death rate \right)_{3}-{diff rate}_{4}$  $\left( diff.rate+death rate \right)_{3}$ – estimate found in “*DP cell differentiation rate*” section (3^rd^ option)  ${diff rate}_{4}$ – estimate found in “*DP cell differentiation rate*” section (4^th^ option)  *Scaling factor: 10^3^* (9,24):  $death rate$ = 1.050 – 0.02 (SP4) – 0.011 (SP8) = 1.019 $\mathrm{da}y^{-1}$  *Scaling factor: 3*10^3^* (9,24):  $death rate$ = 3.140 – 0.061 (SP4) – 0.034 (SP8) = 3.045 $\mathrm{da}y^{-1}$ |
| Number of divisions and time for 1 division, DP cells | $N_{step2}= N_{step1}* 2^{n_{divisions}}$  $1 division time= \frac{residence time}{n_{divisions}}$  $n_{divisions}= {log}_{2}\left( \frac{N\left( DPpre-sel \right)}{N\left( DN3 \right)} \right)= {log}_{2}220=7.78136$  Residence time in DP pre-sel = 1.3 – 2.5 days (mean = 1.9 days) (*see Table S4*)  Time required for 1 division:  *Lower limit of residence time:* 1.3 / 7.78136 = 0.167 days (1 division – 0.167 days)  *Mean value of residence time:* 1.9 / 7.78136 = 0.244 days (1 division – 0.244 days)  *Upper limit of residence time:* 2.5 / 7.78136 = 0.32 days (1 division – 0.32 days) |
| DP cell proliferation rate ($\lambda_{2}$, d^-1^: [3.125; 5.988]) | $proliferation rate=\frac{1}{1 division time}$  *Lower limit of residence time:* 1 / 0.167 = 5.988 $\mathrm{da}y^{-1}$  *Mean value of residence time:* 1 / 0.244 = 4.098 $\mathrm{da}y^{-1}$  *Upper limit of residence time:* 1 / 0.32 = 3.125 $\mathrm{da}y^{-1}$ |
| DP cell differentiation rate  ($\varphi_{4}$, d^-1^: [0.004; 1.930];  $\varphi_{8}$, d^-1^; [0.002; 0.960]) | ${DPss}_{5-6 w.o.}=96.5*{10}^{6}*1.1=106.15*{10}^{6}$ cells (see section “*DN cell differentiation rate*” (1^st^ option) for derivation)  1^st^ option (mouse data, via turnover rate):  $diff. rate+death rate= \frac{DP turnover rate}{{DPss}_{5-6 w.o.}}= \frac{2.3*{10}^{7}}{106.15*{10}^{6}}=0.217 \mathrm{da}y^{-1}$  $diff. rate= 0.217-death rate \left( 0.25 \right)<0$  2^nd^ option (mouse data, via production rate):  $diff.rate to SP4 \left( or SP8 \right) cells= \frac{Production rate of SP4 \left( or SP8 \right) cells}{{DPss}_{5-6 w.o.}}$  Production rate / DP steady-state = differentiation rate from DP to SP4 and SP8:  $diff.rate to SP4 cells=\frac{4.5*{10}^{5}}{106.15*{10}^{6}}=0.0042 \mathrm{da}y^{-1}$  $diff.rate to SP8 cells=\frac{2.5*{10}^{5}}{106.15*{10}^{6}}=0.0024 \mathrm{da}y^{-1}$  3^rd^ option (mouse data scaled to human, via turnover rate):  $diff. rate+death rate= \frac{Turnover rate (scaled for human)}{DP steady-state (human)}$  $DP steady-state (human)$ = 2.2*10^10^ cells (mean value for the 0-to-1 year old age group (range [1.2*10^10^; 3.2*10^10^] (*see Table S2*)))  *Scaling factor: 10^3^* (9,24):  $diff. rate+death rate= \frac{2.3*{10}^{7}*{10}^{3}}{2.2*{10}^{10}}=1.05 \mathrm{da}y^{-1}$  $diff. rate+death rate \left( 1.05 \right)- {death rate}_{1}\left( 0.25 \right)=1.05-0.25=0.8 \mathrm{da}y^{-1}$  ${death rate}_{1}$*-* estimate found in “*DP cell death rate*” section (1^st^ option)  Assuming a 2/1 differentiation ratio for SP4/SP8 (since the SP4/SP8 cell count is approximately 2):  $diff. rate \left( SP4 \right)=0.8*\frac{2}{3}=0.53 \mathrm{da}y^{-1}$  $diff. rate \left( SP8 \right)=0.82*\frac{1}{3}=0.27 \mathrm{da}y^{-1}$  $diff. rate+death rate \left( 1.05 \right)- {death rate}_{2}\left( 0.92 \right)=1.05-0.92=0.13 \mathrm{da}y^{-1}$  ${death rate}_{2}$*-* estimate found in “*DP cell death rate*” section (2^nd^ option)  Assuming a 2/1 differentiation ratio for SP4/SP8 (since the SP4/SP8 cell count is approximately 2):  $diff. rate \left( SP4 \right)=0.13*\frac{2}{3}=0.087 \mathrm{da}y^{-1}$  $diff. rate \left( SP8 \right)=0.15*\frac{1}{3}=0.043\mathrm{da}y^{-1}$  *Scaling factor: 3*10^3^* (9,24):  $diff. rate+death rate= \frac{2.3*{10}^{7}*{3*10}^{3}}{2.2*{10}^{10}}=3.14 \mathrm{da}y^{-1}$  $diff. rate+death rate \left( 3.14 \right)- {death rate}_{1}\left( 0.25 \right)=3.14-0.25=2.89 \mathrm{da}y^{-1}$  ${death rate}_{1}$*-* estimate found in “*DP cell death rate*” section (1^st^ option)  Assuming a 2/1 differentiation ratio for SP4/SP8 (since the SP4/SP8 cell count is approximately 2):  $diff. rate \left( SP4 \right)=2.89*\frac{2}{3}=1.93 \mathrm{da}y^{-1}$  $diff. rate \left( SP8 \right)=2.89*\frac{1}{3}=0.96 \mathrm{da}y^{-1}$  $diff. rate+death rate \left( 3.14 \right)- {death rate}_{2}\left( 0.92 \right)=3.14-0.92=2.22 \mathrm{da}y^{-1}$  ${death rate}_{2}$*-* estimate found in “*DP cell death rate*” section (2^nd^ option)  Assuming a 2/1 differentiation ratio for SP4/SP8 (since the SP4/SP8 cell count is approximately 2):  $diff. rate \left( SP4 \right)=2.22*\frac{2}{3}=1.48 \mathrm{da}y^{-1}$  $diff. rate \left( SP8 \right)=2.22*\frac{1}{3}=0.74 \mathrm{da}y^{-1}$  4^th^ option (mouse data scaled to human, via production rate):  $diff.rate to SP4 \left( or SP8 \right) cells= \frac{Production rate of SP4 \left( or SP8 \right) cells (scaled for human)}{DP steady-state (human)}$  $DP steady-state (human)$ = 2.2*10^10^ cells (mean value for the 0-to-1 year old age group (range [1.2*10^10^; 3.2*10^10^] (*see Table S2*)))    *Scaling factor: 1*10^3^* (9,24):  $diff.rate to SP4 cells=\frac{4.5*{10}^{5}*{10}^{3}}{2.2*{10}^{10}}=0.020 \mathrm{da}y^{-1}$  $diff.rate to SP8 cells=\frac{2.5*{10}^{5}*{10}^{3}}{2.2*{10}^{10}}=0.011 \mathrm{da}y^{-1}$  *Scaling factor: 3*10^3^* (9,24):  $diff.rate to SP4 cells=\frac{4.5*{10}^{5}*{3*10}^{3}}{2.2*{10}^{10}}=0.061 \mathrm{da}y^{-1}$  $diff.rate to SP8 cells=\frac{2.5*{10}^{5}*{3*10}^{3}}{2.2*{10}^{10}}=0.034 \mathrm{da}y^{-1}$ |
| **SP thymocytes** | |
| SP cell egress rate  ($\varepsilon_{4}$, d^-1^: [0.06; 0.23];  $\varepsilon_{8}$, d^-1^: [0.06; 0.22]) | 1^st^ option:  $exit rate= \frac{exported SP cells}{SP cells steady-state}$  Exported SP thymocytes: ~ 1 % of all thymocytes, 1-3 * 10^6^ cells/day (mouse data, (15))  $exit rate= \frac{2*{10}^{6}}{17* {10}^{6}}=0.12 \mathrm{day}^{-1}$  Assuming equal export rates for SP4 and SP8, $exit rate \left( SP4 \right)= exit rate \left( SP8 \right)=0.06 \mathrm{day}^{-1}$  2^nd^ option:  $exit rate= \frac{1}{residence time of SP cells}$  SP4 and SP8 emigrate from the thymus after 4.4 and 4.6 days, respectively (19):  $exit rate \left( SP4 \right)=\frac{1}{4.4}=0.23 \mathrm{day}^{-1}$  $exit rate \left( SP8 \right)=\frac{1}{4.6}=0.22 \mathrm{day}^{-1}$ |
| Number of divisions and time for 1 division, SP cells | For detailed formulae, see “*Number of divisions and time for 1 division, DN cells*” section:  $n_{divisions}= {log}_{2}\left( \frac{N\left( SP \right)}{N\left( DP post-selection \right)} \right)= {log}_{2}2=1$  Residence time for SP ≈ 4.5 days (mouse data, (19)) or 5.4 days (mouse data, (9))  Time required for 1 cell division:  *SP cell residence time = 4.5:* 4.5 / 1 = 4.5 days (1 division – 4.5 days)  *SP cell residence time = 5.4:* 5.4 / 1 = 5.4 days (1 division – 5.4 days) |
| SP cell proliferation rate  ($\lambda_{4}$, d^-1^: [0.19; 0.22];  $\lambda_{8}$, d^-1^: [0.19; 0.22]) | For detailed formulae, “*DN cell proliferation rate*” section  Proliferation rate:  *SP cell residence time = 4.5:* 1 / 4.5 = 0.220 $\mathrm{day}^{-1}$  *SP cell residence time = 5.4:* 1 / 5.4 = 0.185 $\mathrm{day}^{-1}$ |
| SP cell death rate  ($m_{4}$, d^-1^: [0; 0.06];  $m_{8}$, d^-1^: [0; 0.12]) | 1^st^ option (mouse data scaled to human, via turnover rate):  $exit rate+death rate= \frac{Turnover rate (scaled for human)}{SP steady-state (human)}$  $SP4 steady-state (human)$ = 1.14*10^10^ cells (mean value for the 0-to-1 year old age group (range [6.3*10^9^; 1.7*10^10^] (*see Table S2*)))  $SP8 steady-state (human)$ = 4.17*10^9^ cells (mean value for the 0-to-1 year old age group (range [2.3*10^9^; 6.0*10^9^] (*see Table S2*)))  *Scaling factor:3*10^3^* (9,24)  $SP4: exit rate+death rate= \frac{4.5*{10}^{5}*3*{10}^{3}}{1.14*{10}^{10}}=0.12 \mathrm{da}y^{-1}$  $SP4: exit rate+death rate \left( 0.12 \right)- {exit rate}_{1}\left( \left[ 0.06;0.23 \right] \right)=[0;0.06] \mathrm{da}y^{-1}$  ${exit rate}_{1}$*-* estimates found in “*SP cell egress rate*” section  $SP8: exit rate+death rate= \frac{2.5*{10}^{5}*3*{10}^{3}}{4.17*{10}^{9}}=0.18 \mathrm{da}y^{-1}$  $SP8: exit rate+death rate \left( 0.18 \right)- {exit rate}_{1}\left( \left[ 0.06;0.22 \right] \right)=[0;0.12] \mathrm{da}y^{-1}$  ${exit rate}_{1}$*-* estimates found in “*SP cell egress rate*” section |

Supplementary Table 5. Estimates of model parameters for thymus density ($Dens\left( age \right)$) and volume ($Vol\left( age \right)$)

| **Parameter** | **Value** | **SE** | **RSE, %** | **Data** |
| --- | --- | --- | --- | --- |
| $k_{d}$, g*cm^3 -1^*y^-1^ | 0.001 | 2.7*10^-4^ | 27 | (1) |
| $b_{d}$, g*cm^3 -1^ | 1.014 | 0.013 | 1.3 | (1) |
| $k_{vol}$, cm^3^*y^-1^ | 0.056 | 0.031 | 55.4 | (1) |
| $b_{vol}$, cm^3^ | 25.18 | 1.525 | 6.1 | (1) |

Supplementary Table 6. Procedures used to calculate parameter values for simulation scenarios.

| **Simulation scenario description** | **Parameter values** | **Calculation** |
| --- | --- | --- |
| Healthy subjects | Average values of parameters from physiologically plausible parameter sets | - |
| Early-onset involution (subjects with multiple sclerosis) | $EC_{50}=40$ years | Patients with multiple sclerosis (MS) exhibit early-onset thymus involution, since they feature levels of TREC-containing CD4+ and CD8+ T cells which would be equivalent to those found in 30-year older healthy subjects (25). This indicates that thymic output for MS patient can be captured by $EC_{50}$ (the age corresponding to the 50% of the maximum decrease in thymus wet weight) value adjustment.  According to Chiarini et al. results: (26)  1. Blood CD4+ RTE cells (for healthy subjects) = 3.2 cells/uL  2. Blood CD4+ RTE cells (for MS patients prior to fingolimod treatment) = 1.7 cells/uL  $\frac{RTE_{MS}}{RTE_{healthy}}=\frac{1.7}{3.2}=0.53=53\%$ ~ thymic output would be 53% lower in MS patients compared to healthy subjects.  53% decrease in thymic output for 36.9 y.o. patients (average age of patients from Chiarini et al. study (26)) is achieved in case of $EC_{50}=40$ years (thymocyte dynamics model simulation) |
| Early-onset involution and blocked thymic egress (subjects with multiple sclerosis on fingolimod therapy) | $EC_{50}=40$ years  $\varepsilon_{4}=0.011 d^{-1}$  $\varepsilon_{8}=0.012 d^{-1}$ | According to the Chiarini et al. results: (26)  1. Blood CD4+ RTE cells (for MS patients, prior to fingolimod treatment) = 1.7 cells/uL  2. Blood CD4+ RTE cells (for MS patients, after 12-month fingolimod treatment) = 0.26 cells/uL  $\frac{RTE_{MS, f}}{RTE_{MS}}=\frac{0.26}{1.7}=0.15=15\%$ ~ thymic output would be 85% lower after fingolimod treatment compared to MS patients.  $\varepsilon_{4}$ and $\varepsilon_{8}$ estimates are calculated by average value of subsequent parameters ($\varepsilon_{4}: 0.076 d^{-1}*0.15 \sim0.011 \mathrm{day}^{-1};$  $\varepsilon_{8}: 0.082 d^{-1}*0.15 \sim0.012 \mathrm{day}^{-1}$ ) |

Supplementary Table 7. Procedures used to calculate the average threshold of thymic output for fingolimod discontinuation.

| Calculated process | Calculation procedure |
| --- | --- |
| Blood absolute lymphocyte count for Grade 4 lymphopenia according to Common Terminology Criteria for Adverse Events (CTCAE) | < 200 cells/mm^3^ (27) |
| Total absolute lymphocyte count for Grade 4 lymphopenia | - Average blood volume = 5L (28) - % circulating T cells = 2 % (29)   Total absolute lymphocyte count = $\frac{200*{10}^{6}\mathrm{cells}/L* 5 L}{0.02}=5*{10}^{10}\mathrm{cells}$ |
| Contribution of the thymic output into the total lymphocyte count | - Total lymphocyte count for healthy ~25 y.o. men = 7 * 10^11^ cells (22) - Thymic output for healthy 25 y.o. men = 5.5 * 10^8^ cells/day (developed model prediction)   thymic output contribution to the total lymphocyte count = $\frac{5.5*{10}^{8}}{7*{10}^{11}}=0.000786 \mathrm{day}^{-1}=0.0786 {\%}/\mathrm{day}$ |
| Thymic output for Grade 4 lymphopenia (threshold of thymic output for fingolimod discontinuation) | Thymic output for Grade 4 lymphopenia = $5*{10}^{10}*0.000786=3.93*{10}^{7}\mathrm{cells}/\mathrm{day}$ |

Supplementary Table 8. Comparisons of parameters - presently developed model vs. previously published thymus models.

| **Parameter** | **Description** | **BaselineValue** | **Generalized estimates** | **Values from other models** |
| --- | --- | --- | --- | --- |
| $\phi$, cells*day^-1^ | thymocyte precursor inflow rate | 4.8*10^5^ | [10^4^; 8.1*10^7^] | **3.78*10^4^** cells/day (for 10^8^ cell thymus) - *Thomas-Vaslin et al.* model (12,20)  **3.7** cells/day (for 10^8^ cell thymus) – *Moleriu et al.* model (17,20) |
| $\varphi_{1}$, day^-1^ | differentiation rate from DN to DP cells | 0.21 | [0.2066; 0.2168] | **0.137** 1/day – *Sawicka et al.* model (16,20)  **0.3** 1/day – *Ye et al.* model (TN => ITTP) (30)  **0.5** 1/day – *Ye et al.* model (ITTP => DP) (30)  **9.52** 1/day – *Cai et al.* model (DN4 => DP) (31) |
| $\mu_{1}$, day^-1^ | death rate of DN cells | 0.056 | [0.053; 0.063] | **0.06** 1/day - *Moleriu et al.* model (17,20)  **0.22** 1/day – *Cai et al.* model (31) |
| $\mu_{2}$, day^-1^ | death rate of DP cells | 0.5 | [0.487; 0.507] | **0** 1/day (early DP) - *Thomas-Vaslin et al.* model (12,20)  **0.043** 1/day (DP3 (differentiate to SP8)) – *Sinclair et al.* model (18,20)  **0.209** 1/day (DP1) – *Sinclair et al.* model (18,20)  **0.24** 1/day – *Cai et al.* model (31)  **0.263** 1/day (pre-sel. DP) – *Sawicka et al.* model (16,20)  **0.3** 1/day - *Moleriu et al.* model (17,20)  **0.33** 1/day - *Ye et al.* model (30)  **0.3441** 1/day (late-stage DP) - *Thomas-Vaslin et al.* model (12,20)  **0.49** 1/day (DP2) – *Sinclair et al.* model (18,20)  **1.34** 1/day (post-sel. DP) – *Sawicka et al.* model (16,20) |
| $\lambda_{1}$, day^-1^ | proliferation rate of DN cells | 1.67 | [1.627; 1.693] | **0.51** 1/day - *Moleriu et al.* model (17,20)  **1.5** 1/day – *Ye et al.* model (TN) (30)  **9.48** 1/day – *Cai et al.* model (DN4 => DP) (31) |
| $\lambda_{2}$, day^-1^ | proliferation rate of DP cells | 3.125 | [3.106; 3.233] | **0.177** 1/day - *Moleriu et al.* model (17,20)  **1.5** 1/day – *Ye et al.* model (30) |
| $\varphi_{4}$, day^-1^ | differentiation rate from DP to SP4 cells | 0.8 | [0.491; 1.042] | **0.021** 1/day - *Thomas-Vaslin et al.* model (12,20)  **0.03** 1/day - *Moleriu et al.* model (17,20)  **0.039** 1/day – *Sinclair et al.* model (18,20)  **0.04** 1/day – *Ye et al.* model (30)  **0.07** 1/day – *Sawicka et al.* model (16,20) |
| $\varphi_{8}$, day^-1^ | differentiation rate from DP to SP8 cells | 0.3 | [0.233; 0.505] | **0.0046** 1/day - *Thomas-Vaslin et al.* model (12,20)  **0.013** 1/day – *Sinclair et al.* model (18,20)  **0.02** 1/day – *Ye et al.* model (30)  **0.04** 1/day - *Moleriu et al.*model (17,20)  **0.093** 1/day – *Sawicka et al.* model (16,20) |
| $\lambda_{4}$, day^-1^ | proliferation rate of SP4 cells | 0.22 | [0.19; 0.877] | **0** 1/day – *Sinclair et al.* model (18,20)  **0.134** 1/day - *Moleriu et al.* model (17,20)  **0.216** 1/day – *Sawicka et al.* model (16,20)  **0.23** 1/day - *Thomas-Vaslin et al.* model (12,20)  **0.5** 1/day – *Ye et al.* model (30) |
| $\lambda_{8}$, day^-1^ | proliferation rate of SP8 cells | 0.22 | [0.19; 1.338] | **0** 1/day – *Sinclair et al.* model (18,20)  **0.093** 1/day – *Sawicka et al.* model (16,20)  **0.23** 1/day - *Thomas-Vaslin et al.* model (12,20)  **0.238** 1/day - *Moleriu et al.* model (17,20)  **0.5** 1/day – *Ye et al.* model (30) |
| $\mu_{4}$, day^-1^ | death rate of SP4 cells | 0.005 | [0; 0.029] | **0** 1/day - *Thomas-Vaslin et al.* model (12,20)  **0.037** 1/day – *Sinclair et al.* model (18,20)  **0.04** 1/day – *Sawicka et al.* model (16,20)  **0.085** 1/day – *Cai et al.* Model (death+egress) (31)  **0.26** 1/day – *Ye et al.* model (30)  **0.28** 1/day - *Moleriu et al.* model (death+egress) (17,20) |
| $\mu_{8}$, day^-1^ | death rate of SP8 cells | 0.005 | [0; 0.022] | **0** 1/day - *Thomas-Vaslin et al.* model (12,20)  **0.085** 1/day – *Cai et al.* Model (death+egress) (31)  **0.11** 1/day – *Sawicka et al.* model (16,20)  **0.11** 1/day – *Sinclair et al.* model (18,20)  **0.26** 1/day – *Ye et al.* model (30)  **0.56** 1/day - *Moleriu et al.* model (death+egress) (17,20) |
| $\varepsilon_{4}$, day^-1^ | egress rate of SP4 cells | 0.06 | [0.046; 0.084] | **0.07** 1/day – *Ye et al.* model (30)  **0.085** 1/day – *Cai et al.* model (death+egress) (31)  **0.154** 1/day – *Sinclair et al.* model [(18,20)  **0.21** 1/day – *Sawicka et al.* model (16,20)  **0.28** 1/day - *Moleriu et al.* model (death+egress) (17,20)  **0.3664** 1/day - *Thomas-Vaslin et al.* model (12,20) |
| $\varepsilon_{8}$, day^-1^ | egress rate of SP8 cells | 0.06 | [0.036; 0.077] | **0.07** 1/day – *Ye et al.* model (30)  **0.085** 1/day – *Cai et al.* Model (death+egress) (31)  **0.14** 1/day – *Sawicka et al.* model (16,20)  **0.15** 1/day – *Sinclair et al.* model (18,20)  **0.367** 1/day - *Thomas-Vaslin et al.* model (12,20)  **0.56** 1/day - *Moleriu et al.* model (death+egress) (17,20) |

## Supplementary Figures


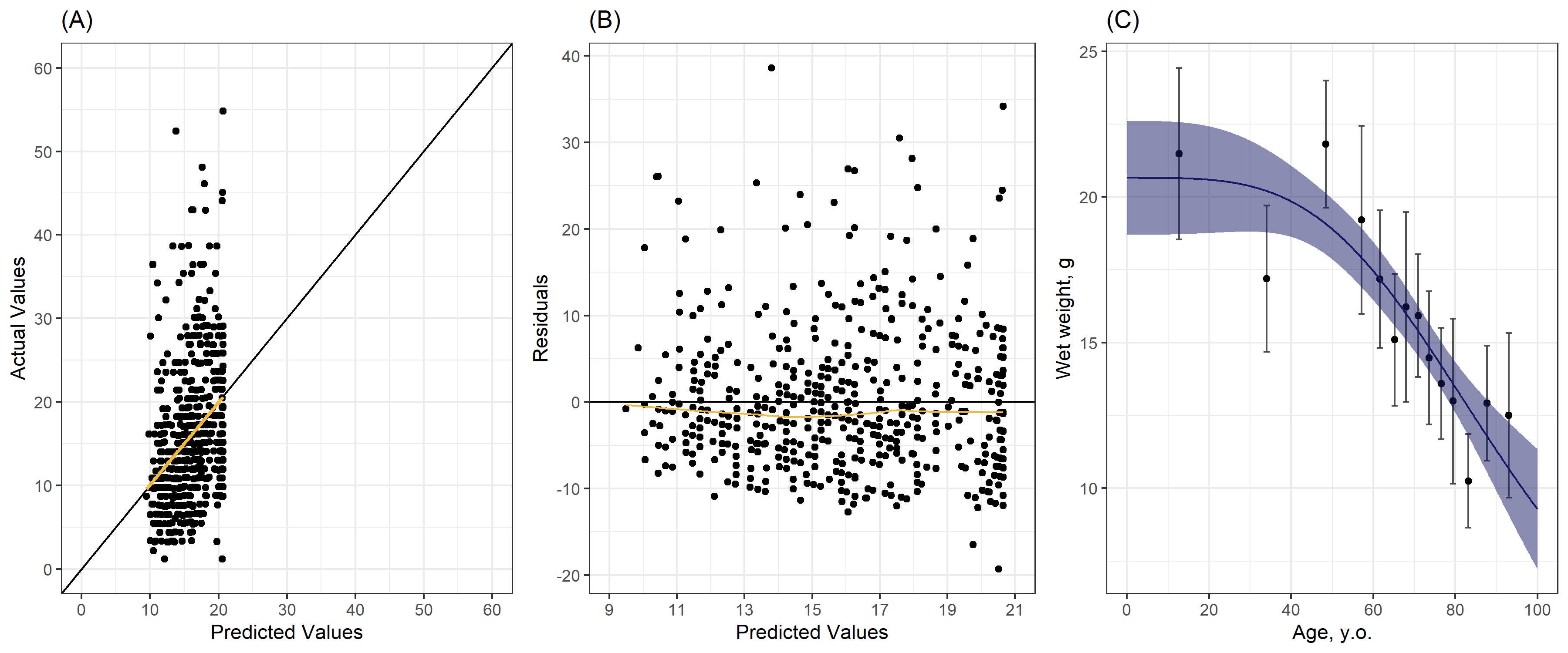


Supplementary Figure 1. Goodness-of-fit plots for nonlinear regressions of thymus wet weights (WW). A – Observed vs. predicted values (yellow line – linear regression line); B – Model residuals vs. predicted values (yellow line – smoothing line obtained by the LOWESS method, data points are expected to scatter around the horizontal zero-line); C – Mean and 95% CI (shaded area) vs. data (dots – mean and 95% CI).


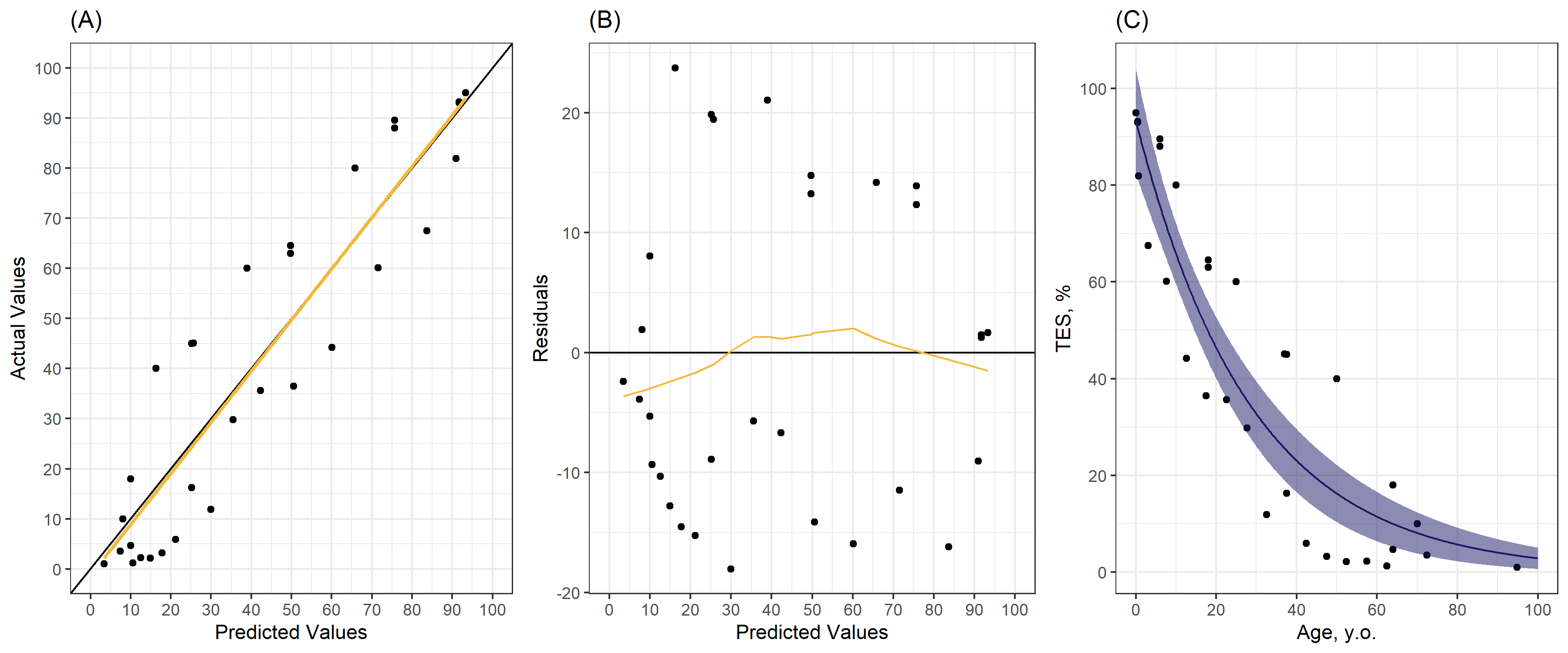


Supplementary Figure 2. Goodness-of-fit plots for nonlinear regressions of the percentage of thymic epithelial space (TES). A – Observed vs. predicted values (yellow line – linear regression line); B – Model residuals vs. predicted values (yellow line – smoothing line obtained by the LOWESS method, data points are expected to scatter around the horizontal zero-line); C – Mean and 95% CI (shaded area) vs. data (dots).


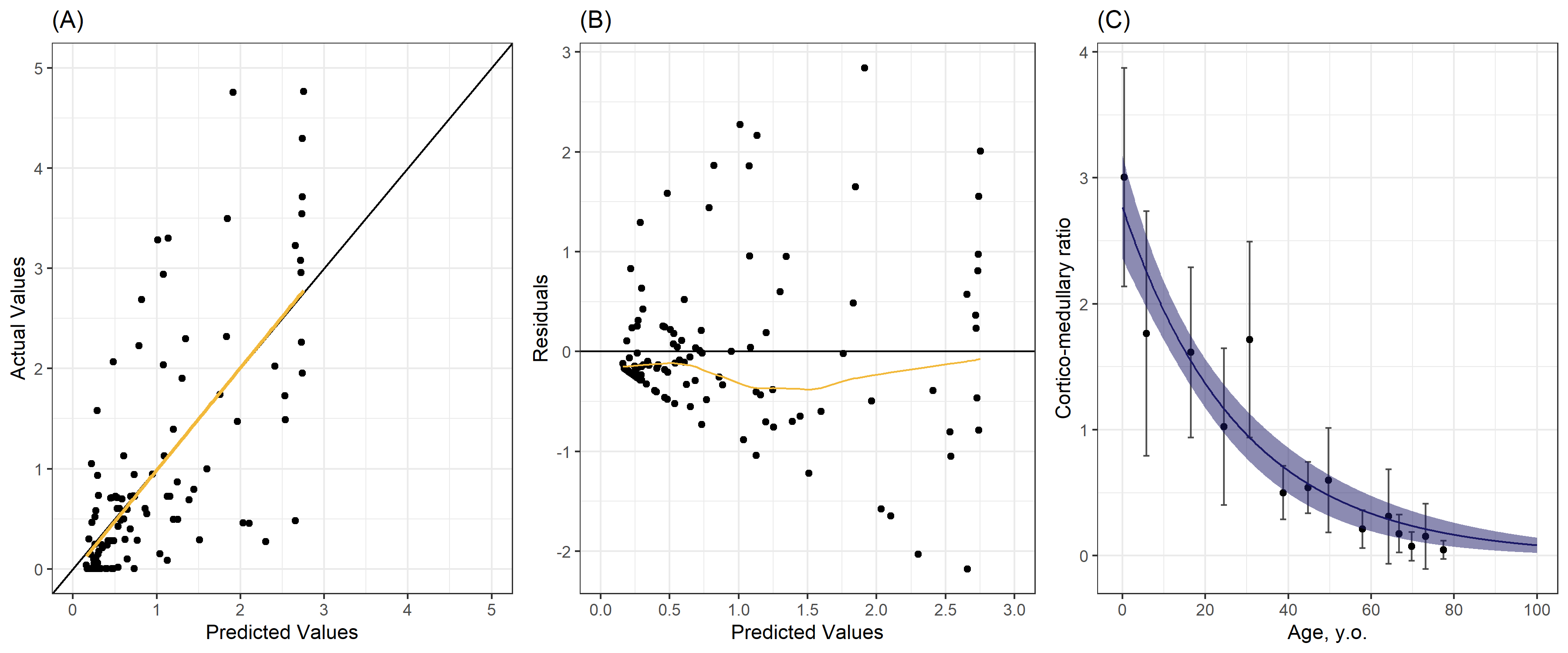


Supplementary Figure 3. Goodness-of-fit plots for nonlinear regressions of cortico-medullary ratios. A – Observed vs. predicted values (yellow line – linear regression line); B – Model residuals vs. predicted values (yellow line – smoothing line obtained by the LOWESS method, data points are expected to scatter around the horizontal zero-line); C – Mean and 95% CI (shaded area) vs. data (dots – mean and 95% CI).


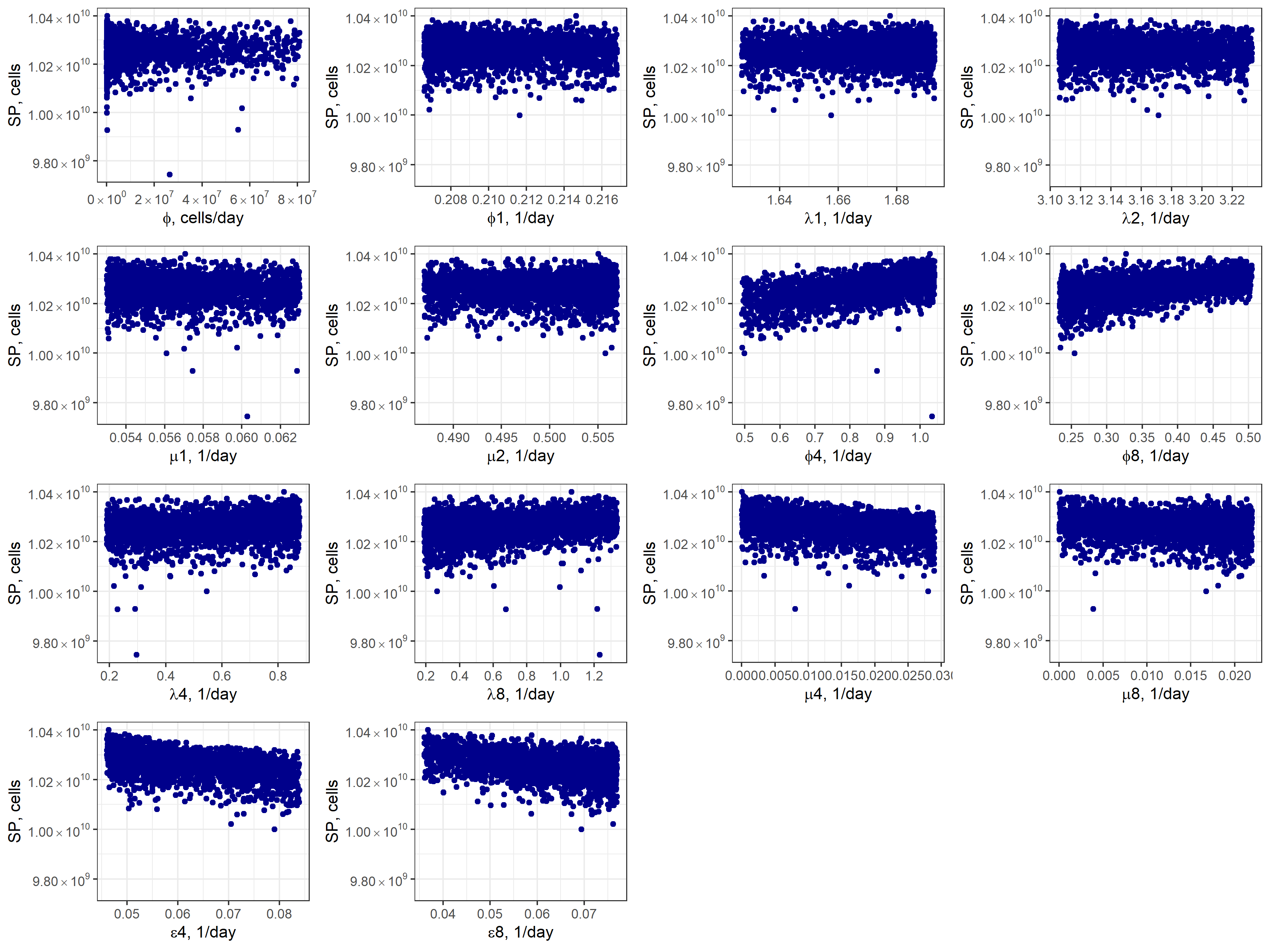


Supplementary Figure 4. Relationship between model output (SP cell counts) and model parameter values based on a physiologically plausible parameter sets (n = 3,474).


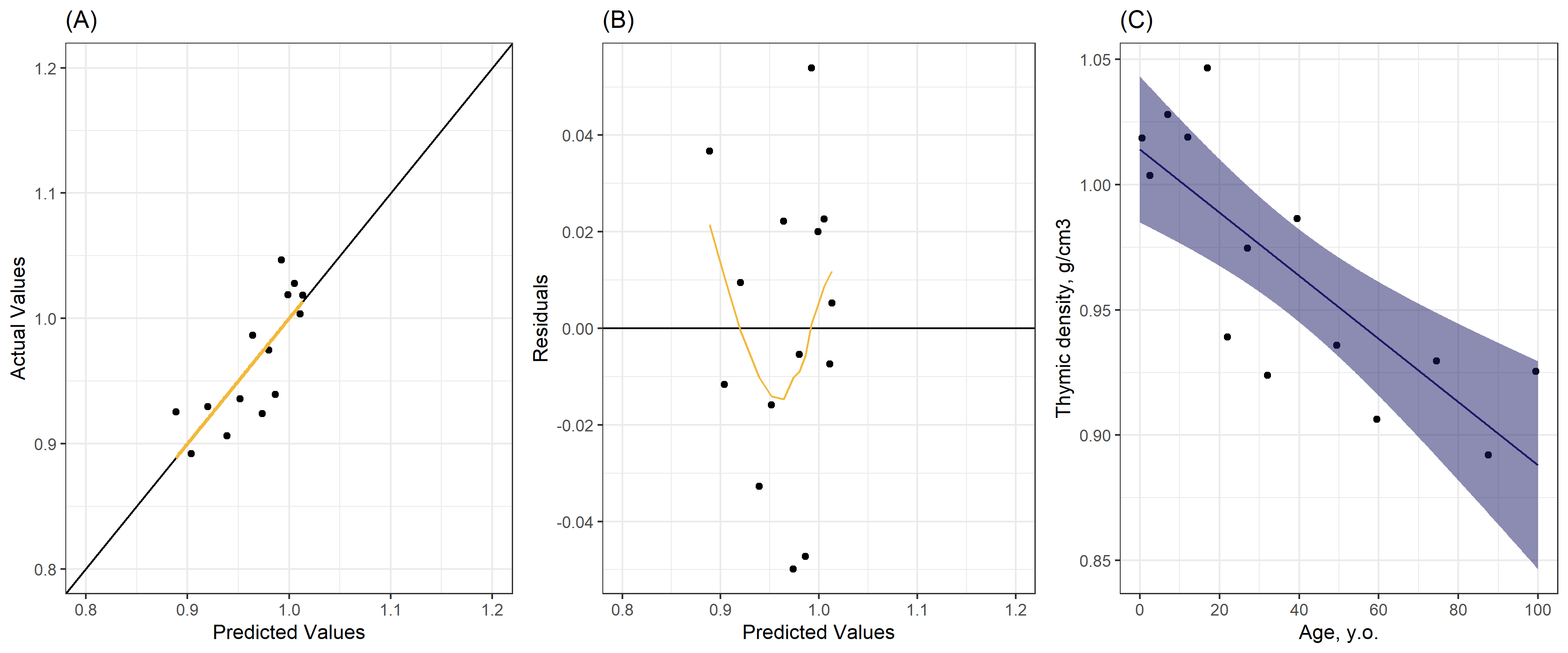


Supplementary Figure 5. Goodness-of-fit plots for linear regression of thymus densities. A – Observed vs. predicted values (yellow line – linear regression line); B – Model residuals vs. predicted values (yellow line – smoothing line obtained by the LOWESS method, data points are expected to scatter around the horizontal zero-line); C – Mean and 95% CI (shaded area) vs. data (dots).


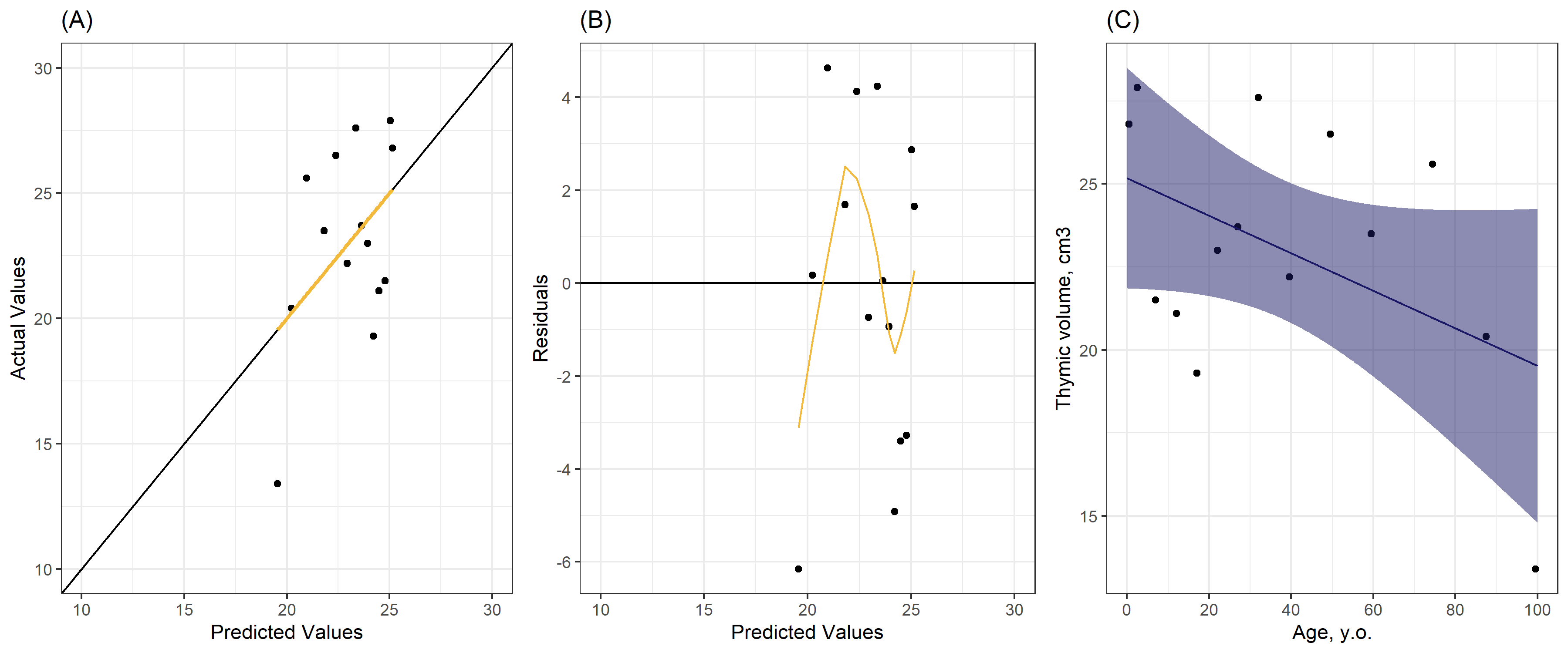


Supplementary Figure 6. Goodness-of-fit plots for linear regression of thymus volumes. A – Observed vs. predicted values (yellow line – linear regression line); B – Model residuals vs. predicted values (yellow line – smoothing line obtained by the LOWESS method, data points are expected to scatter around the horizontal zero-line); C – Mean and 95% CI (shaded area) vs. data (dots).

# References

1. STEINMANN GG, KLAUS B, MÜLLER-HERMELINK HK. The Involution of the Ageing Human Thymic Epithelium is Independent of Puberty. Scand J Immunol. 1985 Nov 1;22(5):563–75.

2. Bertho JM, Demarquay C, Moulian N, Van Der Meeren A, Berrih-Aknin S, Gourmelon P. Phenotypic and Immunohistological Analyses of the Human Adult Thymus: Evidence for an Active Thymus during Adult Life. Cell Immunol. 1997 Jul 10;179(1):30–40.

3. Park JE, Botting RA, Domínguez Conde C, Popescu DM, Lavaert M, Kunz DJ, et al. A cell atlas of human thymic development defines T cell repertoire formation. Science. 2020 Feb 21;367(6480):eaay3224.

4. Varas A, Jiménez E, Sacedón R, Rodríguez-Mahou M, Maroto E, Zapata AG, et al. Analysis of the Human Neonatal Thymus: Evidence for a Transient Thymic Involution. J Immunol. 2000 Jun 15;164(12):6260–7.

5. Lavaert M, Valcke B, Vandekerckhove B, Leclercq G, Liang KL, Taghon T. Conventional and Computational Flow Cytometry Analyses Reveal Sustained Human Intrathymic T Cell Development From Birth Until Puberty. Front Immunol [Internet]. 2020;11. Available from: https://www.frontiersin.org/articles/10.3389/fimmu.2020.01659

6. Fujii Y, Okumura M, Yamamoto S. Flow Cytometric Study of Lymphocytes Associated with Thymoma and Other Thymic Tumors. J Surg Res. 1999 Apr 1;82(2):312–8.

7. Takeuchi Y, Fujii Y, Okumura M, Inada K, Nakahara K, Matsuda H. Characterization of CD4+ Single Positive Cells That Lack CD3 in the Human Thymus. Cell Immunol. 1993 Oct 15;151(2):481–90.

8. Weerkamp F, de Haas EFE, Naber BAE, Comans-Bitter WM, Bogers AJJC, van Dongen JJM, et al. Age-related changes in the cellular composition of the thymus in children. J Allergy Clin Immunol. 2005 Apr 1;115(4):834–40.

9. Krueger A, Ziętara N, Łyszkiewicz M. T Cell Development by the Numbers. Trends Immunol. 2017 Feb 1;38(2):128–39.

10. Krueger A. Thymus Colonization: Who, How, How Many? Arch Immunol Ther Exp (Warsz). 2018 Apr 1;66(2):81–8.

11. Paul WE. Fundamental immunology. 5th ed. Philadelphia: Lippincott Williams & Wilkins; 2003.

12. Thomas-Vaslin V, Altes HK, de Boer RJ, Klatzmann D. Comprehensive Assessment and Mathematical Modeling of T Cell Population Dynamics and Homeostasis. J Immunol. 2008 Feb 15;180(4):2240–50.

13. Egerton M, Shortman K, Scollay R. The kinetics of immature murine thymocyte development in vivo. Int Immunol. 1990 Jun 1;2(6):501–7.

14. Egerton M, Scollay R, Shortman K. Kinetics of mature T-cell development in the thymus. Proc Natl Acad Sci. 1990 Apr 1;87(7):2579–82.

15. Yates A. Theories and Quantification of Thymic Selection. Front Immunol [Internet]. 2014;5. Available from: https://www.frontiersin.org/articles/10.3389/fimmu.2014.00013

16. Sawicka M, Stritesky G, Reynolds J, Abourashchi N, Lythe G, Molina-Paris C, et al. From pre-DP, post-DP, SP4, and SP8 Thymocyte Cell Counts to a Dynamical Model of Cortical and Medullary Selection. Front Immunol [Internet]. 2014;5. Available from: https://www.frontiersin.org/articles/10.3389/fimmu.2014.00019

17. Moleriu RD, Zaharie D, Moatar-Moleriu LC, Gruia AT, Mic AA, Mic FA. Insights into the mechanisms of thymus involution and regeneration by modeling the glucocorticoid-induced perturbation of thymocyte populations dynamics. J Theor Biol. 2014 May 7;348:80–99.

18. Sinclair C, Seddon B. Overlapping and Asymmetric Functions of TCR Signaling during Thymic Selection of CD4 and CD8 Lineages. J Immunol. 2014 Jun 1;192(11):5151–9.

19. McCaughtry TM, Wilken MS, Hogquist KA. Thymic emigration revisited. J Exp Med. 2007 Oct 1;204(11):2513–20.

20. Robert PA, Kunze-Schumacher H, Greiff V, Krueger A. Modeling the Dynamics of T-Cell Development in the Thymus. Entropy. 2021;23(4).

21. Pénit C, Lucas B, Vasseur F. Cell expansion and growth arrest phases during the transition from precursor (CD4-8-) to immature (CD4+8+) thymocytes in normal and genetically modified mice. J Immunol. 1995;154(10):5103–13.

22. Cosgrove J, Hustin LSP, de Boer RJ, Perié L. Hematopoiesis in numbers. Trends Immunol. 2021 Dec 1;42(12):1100–12.

23. Hsu HC, Zhang HG, Li L, Yi N, Yang PA, Wu Q, et al. Age-related thymic involution in C57BL/6J × DBA/2J recombinant-inbred mice maps to mouse chromosomes 9 and 10. Genes Immun. 2003 Sep 1;4(6):402–10.

24. Rangarajan A, Weinberg RA. Comparative biology of mouse versus human cells: modelling human cancer in mice. Nat Rev Cancer. 2003 Dec 1;3(12):952–9.

25. Hug A, Korporal M, Schröder I, Haas J, Glatz K, Storch-Hagenlocher B, et al. Thymic Export Function and T Cell Homeostasis in Patients with Relapsing Remitting Multiple Sclerosis1. J Immunol. 2003 Jul 1;171(1):432–7.

26. Chiarini M, Sottini A, Bertoli D, Serana F, Caimi L, Rasia S, et al. Newly produced T and B lymphocytes and T-cell receptor repertoire diversity are reduced in peripheral blood of fingolimod-treated multiple sclerosis patients. Mult Scler J. 2015 May 1;21(6):726–34.

27. Common Terminology Criteria for Adverse Events (CTCAE) v5.0 [Internet]. Available from: https://ctep.cancer.gov/protocolDevelopment/electronic_applications/docs/CTCAE_v5_Quick_Reference_8.5x11.pdf

28. Starr C, Taggart R. Biology: The Unity and Diversity of Life [Internet]. Wadsworth Publishing Company; 1998. (Books in the Brooks/Cole biology series). Available from: https://books.google.ru/books?id=t7IXAQAAMAAJ

29. Ganusov VV, De Boer RJ. Do most lymphocytes in humans really reside in the gut? Trends Immunol. 2007 Dec 1;28(12):514–8.

30. Ye P, Kirschner DE. Reevaluation of T Cell Receptor Excision Circles as a Measure of Human Recent Thymic Emigrants1. J Immunol. 2002 May 15;168(10):4968–79.

31. Cai AQ, Landman KA, Hughes BD, Witt CM. T cell development in the thymus: From periodic seeding to constant output. J Theor Biol. 2007 Nov 21;249(2):384–94.
